# Decadal monitoring reveals an increase in *Vibrio* spp. concentrations in the Neuse River Estuary, North Carolina, USA

**DOI:** 10.1371/journal.pone.0215254

**Published:** 2019-04-23

**Authors:** Brett Froelich, Raul Gonzalez, Denene Blackwood, Kellen Lauer, Rachel Noble

**Affiliations:** The University of North Carolina at Chapel Hill, Institute of Marine Sciences; Morehead City, NC, United States of America; Nitte University, INDIA

## Abstract

A decade long study was conducted to investigate the ecological, biological, and temporal conditions that affect concentrations of *Vibrio* spp. bacteria in a well-studied lagoonal estuary. Water samples collected from the Neuse River Estuary in eastern North Carolina from 2004–2014 (with additional follow-up samples from Fall of 2018) were analyzed to determine *Vibrio spp*. concentrations, as well as the concentrations of inorganic and organic nutrients, fecal indicator bacteria, phytoplankton biomass, and a wide range of other physio-chemical estuarine parameters. A significant increase in *Vibrio* spp. was observed to occur in the estuary over the examined period. Strikingly, over this long duration study period, this statistically significant increase in total culturable *Vibrio* spp. concentrations does not appear to be correlated with changes in salinity, temperature, or dissolved oxygen, the three most commonly cited influential factors that predict estuarine *Vibrio* spp. abundance. Furthermore, shorter term (~3 years) data on specific *Vibrio* species (*V*. *vulnificus* and *V*. *parahaemolyticus*)show that while *Vibrio* spp. are increasing overall as a genus, the numbers of some key potentially pathogenic species are decreasing as a part of the total population, further supporting the concept that quantification of the entire genus is not a worthwhile use of resources toward predicting levels of specific potentially pathogenic species of public health concern. The significant increase in this concentration of *Vibrio* spp. in the studied estuary appears to be related to nitrogen and carbon in the system, indicating a continued need for further research.

## Introduction

Bacteria in the genus *Vibrio* exhibit a great deal of variation, both phenotypically and genotypically. Most bacteria of the *Vibrio* genus are important aquatic ecosystem members that can be found in fresh, brackish, and marine waters, often with strong, species-specific salinity preferences [1–3]. *Vibrio* are fast-growing, with some species capable of doubling in less than ten minutes, and are therefore able to take rapid advantage of pulses of nutrient or shifts in meteorological conditions [4,5]. *Vibrio* spp. are ubiquitous across aquatic environments and over longer time scales they are biogeochemically important members of mesohaline estuarine environments. While most *Vibrio* spp. are not pathogenic, there exist several species that are pathogenic to humans, fish, eels, shellfish, or other species [6–10]. The increasing number of infections caused by *Vibrio* spp., especially *Vibrio vulnificus*, has generated great a deal of attention and research. Furthermore, *Vibrio* spp. play important roles in ecosystem function and organismal population dynamics, participating in nitrogen fixation, chitin degradation, and metabolism of algal polysaccharides [11–13]. Other well-studied species serve as symbionts, living inside squid or other organisms and functioning as the source of luminescence in light organs and can be important members of biofilms and macroalgal associations, while still other *Vibrio* sp. are capable of degrading petroleum [14,15]. While often monitored for short term changes, there is far less information on the long-term shifts in *Vibrio* spp. populations.

The *Vibrio* spp. found along estuaries and coasts, are of particular interest. Coastal areas offer resources, job opportunity, and the option of recreation and international trade routes [16]. Coastal regions are highly populated worldwide, and US statistics show that populations at the coasts of southeastern states grew 58%, between 1980 and 2003, a rate roughly double that of the rest of the nation [17]. Migration was highest in Florida, Georgia, and North Carolina (NC) [17]. The Neuse River Estuary (NRE) of NC provides extensive commercial and recreational opportunities to residents and visitors while simultaneously serving as the drain for one of the highest producing, including livestock production, and rapidly growing watersheds in the state [18]. The high agricultural, industrial, and urban use of the NRE contributes to a variety of anthropogenic inputs, and the estuary is classified as eutrophic [18].

There have been almost three decades of monitoring and research conducted on the NRE, culminating in numerous peer-reviewed publications about its eutrophic status, discharge characteristics, phytoplankton response to major storms and hypoxia/anoxia dynamics [18–21]. The estuary is an intersection of a large, growing human population, and changing water quality and thus serves as a sentinel for other temperate estuaries worldwide.

While short-term changes in the estuary, such as a nutrient pulse or an extreme heat wave, can contribute to the concentration of *Vibrio* spp. in the context of “Vibrio blooms” [3,22] so too can slow but significant long-term changes also cause alterations in bacterial populations. For example, *Vibrio* spp. are highly correlated with water temperature, and population booms are observed with both short-term upshifts in temperature, such as occur during summer months, as well as long-term temperature shifts associated with climate change [3,23–29]. However, the overall concentrations of the genus *Vibrio* appear to be quite different than those observed for specific species of interest such as *V*. *parahaemolyticus*. Therefore, examination of the genus sometimes can mask important species dynamics. Even though this is true, there are few, if any, long term studies that examined the response of the entire genus over longer periods of time (e.g. decades). This is important for the understanding the potential for future emergence of members of the genus as important contributors to both human and animal disease, and in the context of climate change.

In this study, total *Vibrio* spp. concentrations along with a wide range of estuarine ecosystem, physical, chemical and environmental parameters were monitored along the NRE for ten years (2004–2014. Other *Vibrio* species, specifically human pathogens, were also monitored for four years of the study. The objectives of the analyses were to 1) assess longer-term responses to often cited drivers of *Vibrio* spp. populations, including temperature and salinity, 2) identify additional chemical and biological parameters driving *Vibrio* spp. population change, and 3) examine the relationships of minor level factors in *Vibrio* spp. abundance. Previous studies over shorter time scales have shown that *Vibrio* spp. respond to changes in salinity and temperature [3,4,26,29–39]. This accounts for the seasonal changes, and for the well-documented long-term global climate changes, including warmer weather and extreme events (such as floods, storms, and heat-waves) [25]. A significant increase in *Vibrio* spp. concentrations were observed in the decadal study, but the long-term increases are caused by neither salinity nor temperature increases, as would be expected. Other estuarine factors are reported as correlating with this long-term increase in *Vibrio* spp. concentrations in the NRE.

## Materials and methods

### Study location and period

**Fig 1:**
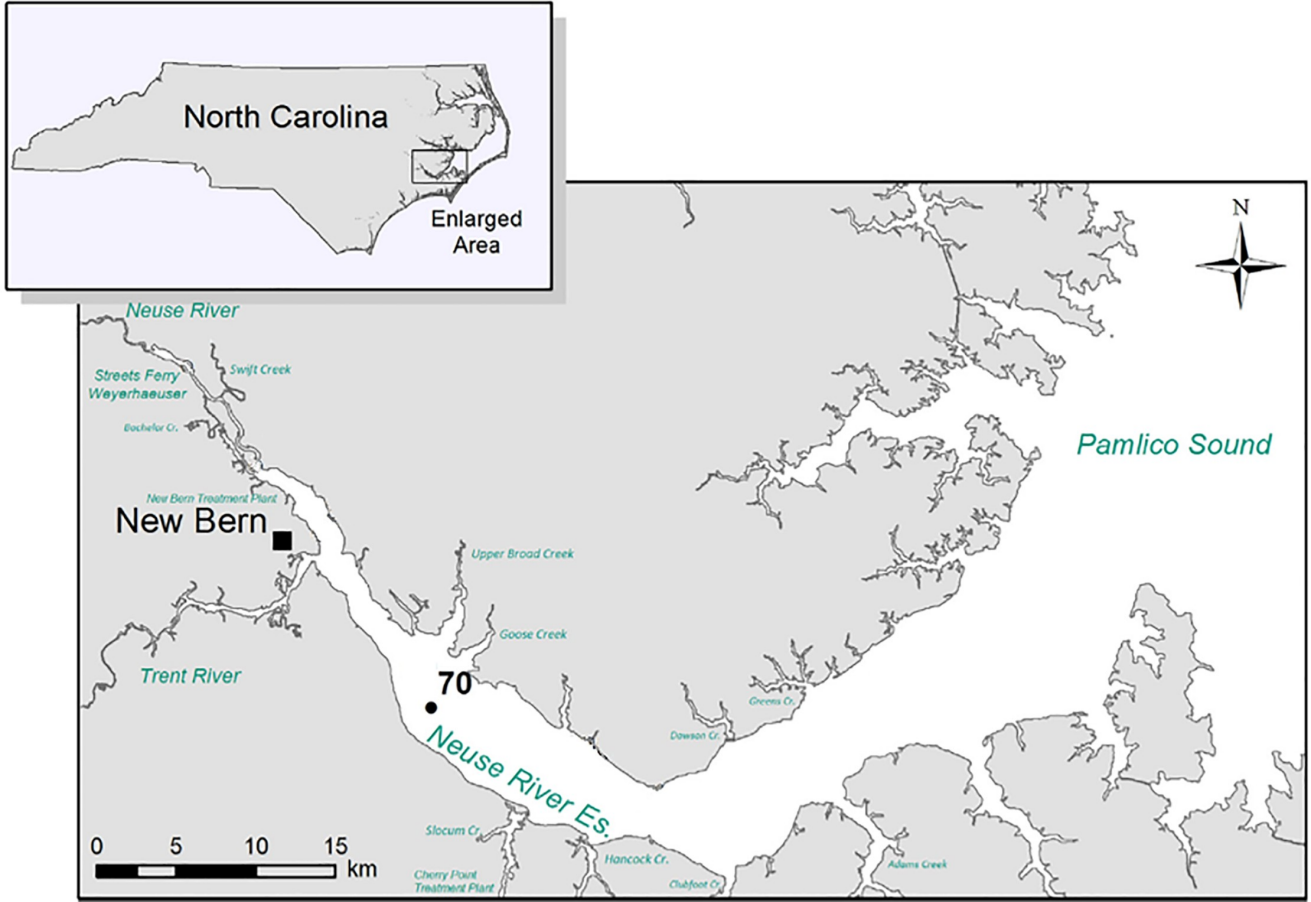
Study region with ModMon sampling stations.

The NRE (Fig 1), located in eastern NC, USA, is a well-described, lagoonal estuary, with wind-driven mixing characteristics and minimal tidal influence due to the protection offered by the proximal Pamlico Sound. Being broad and shallow (generally less than 10 feet in depth), the estuary flow and mixing is dominated by wind and river input [40]. This estuary has been monitored since 1994 through the NRE Modeling and Monitoring program (ModMon, https://goo.gl/BERvPB). For the current project, samples were collected from surface water at one ModMon station (Station 70 Fig 1) along the estuary between June 24, 2004 and October 27^th^, 2014. Sampling occurred approximately every two weeks during warmer periods (between April through November), or every 4 weeks from December through March. This sampling scheme resulted in 245 individual samples collected over the ten-year period. Water was collected via a weighted hose and diaphragm pump into acid washed polypropylene sampling containers. Surface samples were collected at an approximate depth of 0.5 m below the surface [41].

### Measurement of environmental parameters

Concomitant to water sample collection, measurements of water temperature (TEMP), salinity (SAL), dissolved oxygen (DO), pH, turbidity (TURB), and chlorophyll A (CHL) were conducted using a calibrated YSI 6600 multiparameter sonde (Yellow Springs Instruments, Yellow Springs, OH). Water samples were kept in a cooler at ambient temperature and transported to the laboratory within 6 h of collection for immediate analysis.

Water samples were vacuum filtered through precombusted (500°C) 25mm glass fiber filters (GF/F), filtrates were stored in scintillation vials at -20°C. Colored dissolved organic matter (CDOM) was measured on a TD-700 fluorometer (Turner Designs, San Jose, CA). Dissolved organic carbon (DOC) was recorded with a TOC-5000A total organic carbon analyzer (Shimadzu, Pleasanton, CA). Dissolved inorganic carbon (DIC) was measured using HCl acidification followed by analysis on the TOC-5000A total organic carbon analyzer (Shimadzu). The concentration of nitrate and nitrite (NOX) was measured with a QuikChem 8000 flow injection analyzer (Lachat/Zellweger Analytics, Loveland, CO), using method FIA 31-107-04-1-C and ammonium (NH4) was measured using method FIA 31-107-06-1-A/B, total dissolved nitrogen (TDN) used method FIA 31-107-04-03-B as described in Peierls et al [42]. Dissolved inorganic nitrogen (DIN) was calculated by the sum of NOX and NH4. Dissolved organic nitrogen (DON) was calculated by subtracting DIN from TDN. Orthophosphate (PO4) was measured using method FIA 31-115-01-1-F/G.

Total suspended solids (TSS) were measured by vacuum filtering water via laboratory available vacuum water through a 0.7 μm pore size, pre-dried 25mm glass fiber filter until the filter was visibly discolored or 50 ml had been filtered, whichever was greatest. Filters were oven-dried until all moisture was evaporated, and the filter weighed to determine TSS concentrations. TSS results were reported in mg/L.

Particulate organic carbon (POC) and nitrogen (PN) were measured via elemental analysis, as described in Paerl et al [18]. One hundred ml of water was filtered through a precombusted 25mm diameter GF/F filter. Carbonates were removed via vapor phase acidification with HCl. Once dry, filters were rolled up inside tin disks and combusted in a 2400 Series II CHNS/O analyzer (Perkin-Elmer).

### Bacterial quantification

Water samples were diluted with phosphate buffered saline (PBS) and vacuum filtered through 0.45 μm pore size cellulose fiber filters (Pall, Port Washington, NY). Filters were incubated on Thiosulfate-Citrate-Bile Salts-Sucrose (TCBS, Beckton Dickinson) agar at 35°C for 24 hours. For samples collected on August 15^th^, 2011 and onward, additional filters were also placed on ChromAgar *Vibrio* (ChromAGAR, Paris, France) and incubated at 37° for 24 hours. *Vibrio* spp. concentrations were determined by counting the total number of visible yellow and green colonies that exhibited relief from the plate surface from TCBS, adjusting for dilution, and expressing as colony forming units (cfu) per 100 ml. TCBS media manufacturers changed over the course of the study, but all TCBS formulations always adhered to the Bacteriological Analytical Manual [43], and the directions were followed. It should be noted that no recovery testing between batches of TCBS was performed, and thus possible artifacts could exist from changing brands of TCBS or from a manufacturer altering the sources of ingredients. While recipes and methods were standardized to minimize these artifacts, it is impossible to determine if any exist. An assumed concentration of *V*. *vulnificus* was determined by collecting 10 dark blue colonies from ChromAGAR *Vibrio*, and subjecting them to PCR confirmation utilizing the *vvhA* gene as confirmatory for *V*. *vulnificus* as in Warner and Oliver [44].

Total coliform (TC) and *E*. *coli* concentrations (EC) were determined using the Colilert-18® defined substrate technology using the Quanti-Tray 2000 system (IDEXX Laboratories, Westbrook, ME). Trays were incubated for 18-22h at 35°C and most probable number (MPN) was calculated based on aggregate numbers of large and small wells as indicated in the manufacturer’s instructions. Concentrations were reported as MPN per 100 ml.

### Statistical analyses

Station 70S is in the geographic middle of the estuary, is mesohaline, and historically demonstrates fluctuations in both total *Vibrio* spp. concentrations, environmental parameter ranges (making it useful for correlative modeling approaches), and the location of theCHL maximum during certain periods over the time of this study [45]. An alpha of 0.05 was used for all statistical tests. The detection limit for *Vibrio* spp. is 0.5 cells per 100 ml, and the detection limit for fecal indicator bacteria using the Quanti-tray 2000 system is 10 cells per 100 ml. Microbial data were log-transformed prior to analysis. Non-detectable microbial results were given a value of 1 cell per 100 ml. (i.e. 0 log). A Grubb’s outlier test found no significant outliers at the 0.05 level. The *Vibrio* spp. data were log transformed, reducing skewness from 9.16 to -0.48 and kertosis from 91.59 to -0.07.

Yearly periods were analyzed using one-way ANOVA with Tukey post-test. Time series analysis used the monthly mean and produced a seasonal autoregressive integrated moving average (ARIMA) model with order (1,1,1) and seasonal (1,1,1) terms. Six-year groupings were analyzed with a two-way (month and period) ANOVA with Bonferroni post-testing. Ten-year monthly averages were constructed using recorded and predicted values from the ARIMA model projection. Non-linear fit of salinity and *Vibrio* spp. abundance was found using a two-phase exponential associate equation and the Levenberg-Marquardt iteration algorithm. Non-linear fit of salinity over the course of the study was found using a LogNormal equation and the Levenberg-Marquardt iteration algorithm until a fit was converged.

All statistics were calculated using OriginPro 2018 (OriginLab Co., Northampton, MA) except for the principal component analysis and the ARIMIA model, which were performed using JMP 13.0 (SAS Co., Cary, NC).

## Results

### *Vibrio* spp. increase significantly over 10-year period

**Fig 2:**
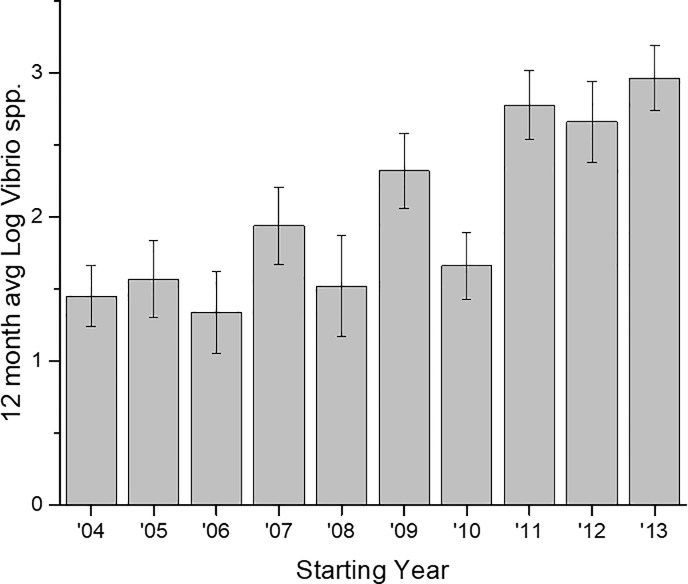
12 month mean of log *Vibrio* spp. at station 70S. Each 12-month period is from June–May. Labels indicate the year that the 12-month period began.

**Fig 3:**
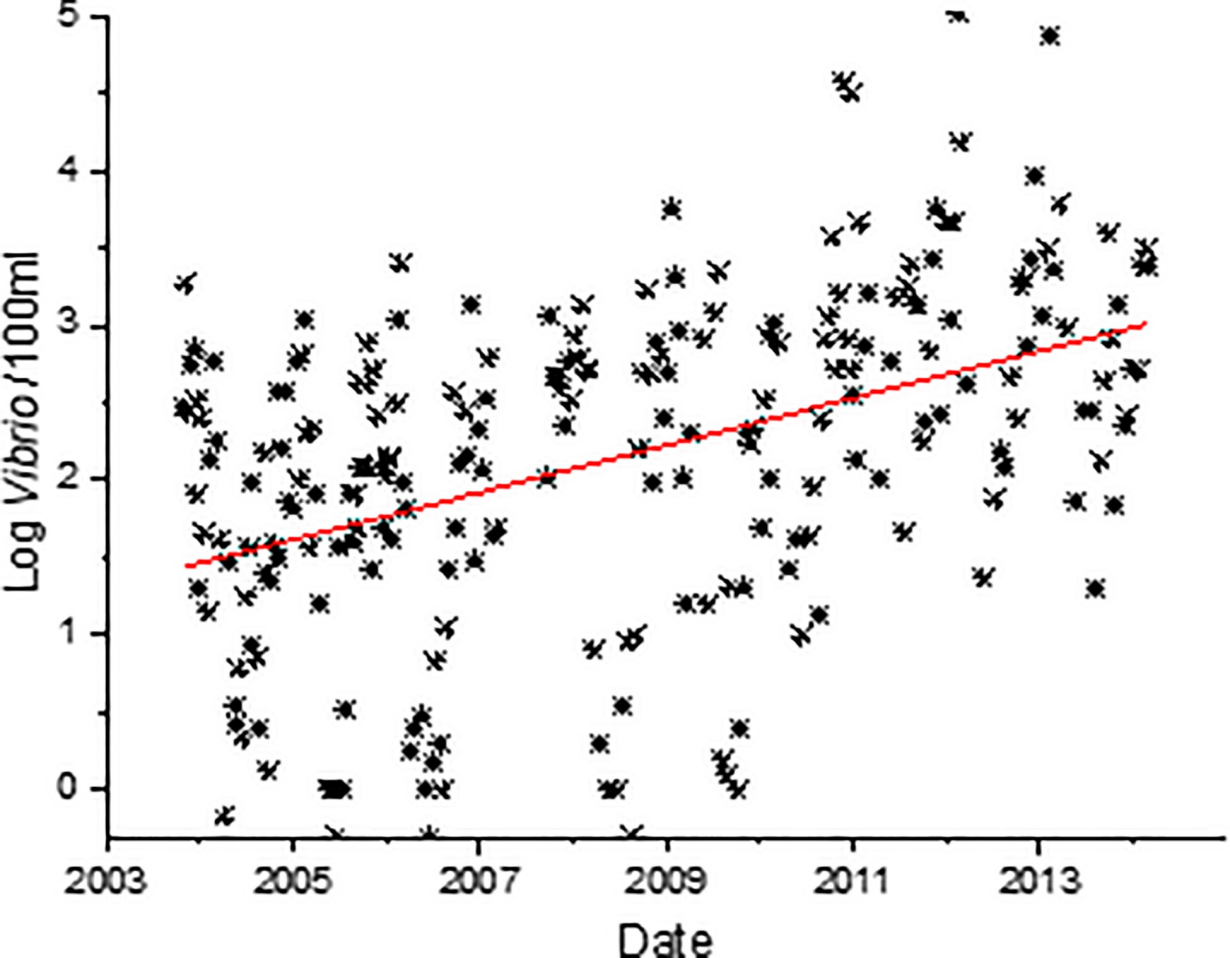
*Vibrio* spp. concentrations at each sampling date for station 70S. Line is the linear regression of the data (r^2^ = 0.20, p<0.0001).

Each 12-month period (June–May) of *Vibrio* spp. abundance data, was averaged (Fig 2), and significant differences among years were observed (p<0.00001). *Vibrio* spp. concentrations in years 2011, 2012, and 2013 were significantly higher than 2006; 2011 and 2013 were also significantly greater than years 2004, and 2008; and 2013 was also significantly greater than 2005, 2007, and 2009 (p<0.05, Fig 2). A weak but significant linear increase in the concentration of *Vibrio* spp. over time at station 70S is shown in Fig 3 (r^2^ = 0.20, p<0.0001).

**Fig 4:**
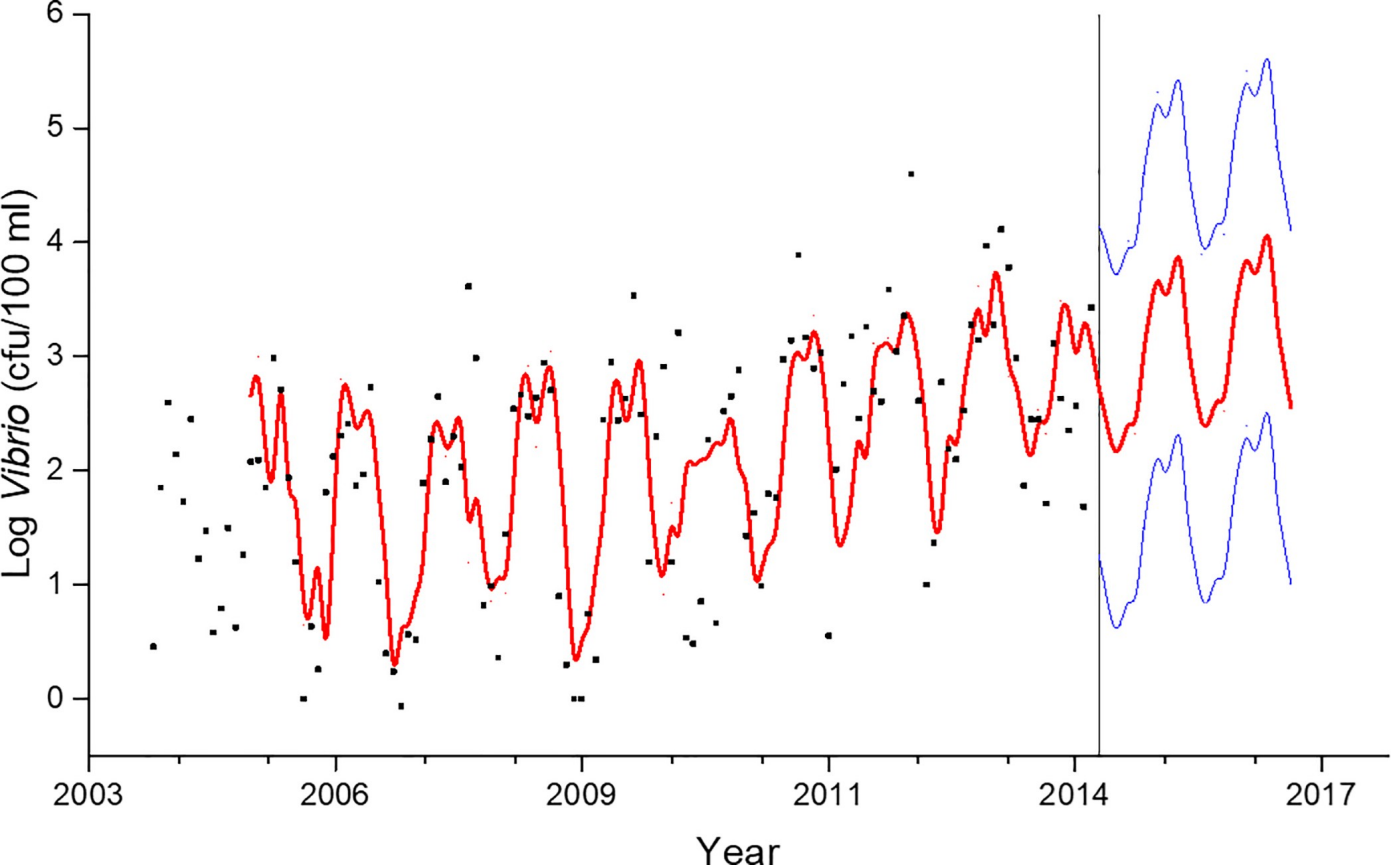
Seasonal ARIMA model (1,1,1)(1,1,1) of mean monthly log *Vibrio* spp. data at station 70S. Dots are actual measurements, red line represents modeled abundance, and blue lines are the 95% confidence interval.

**Fig 5:**
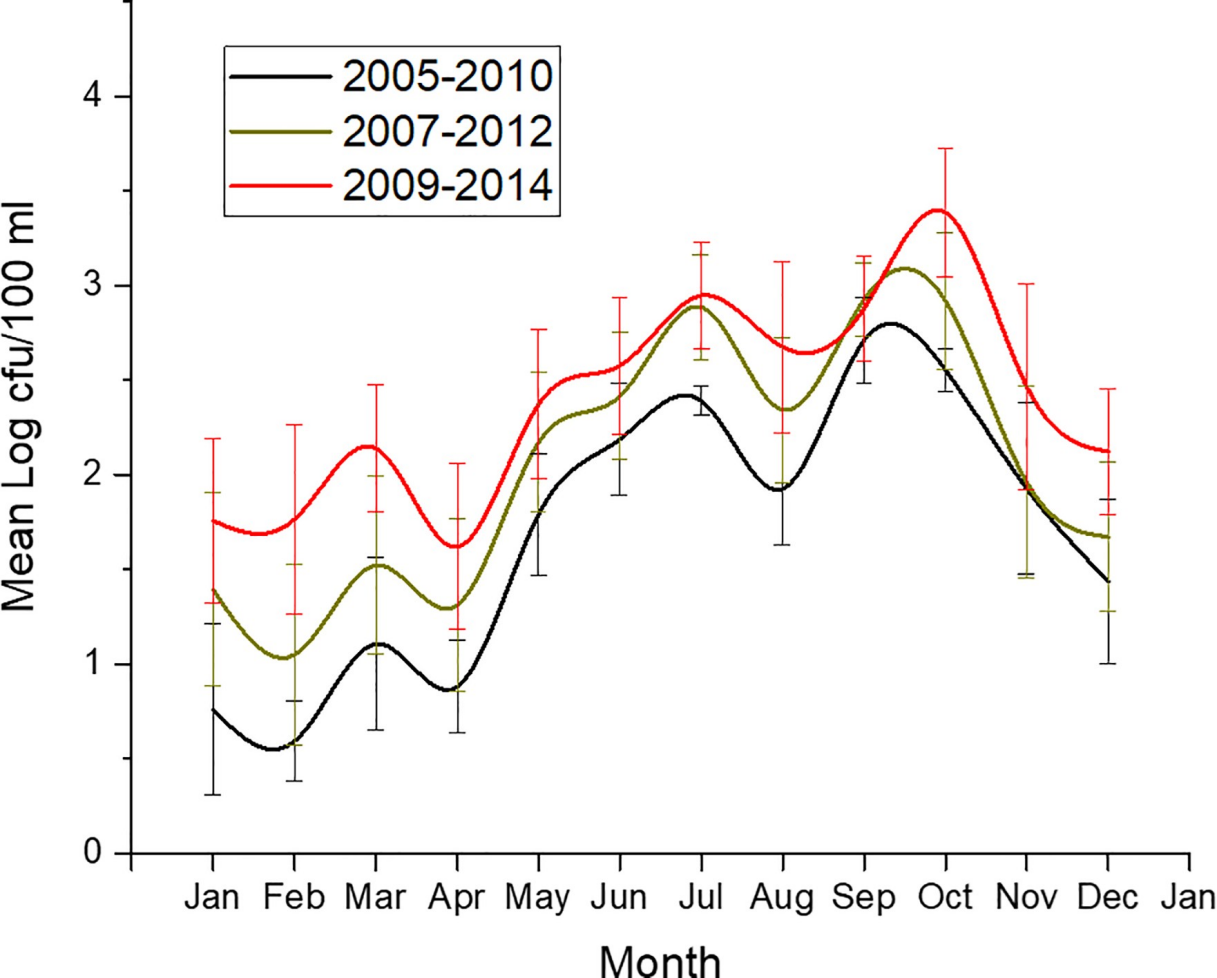
Monthly six-year average of *Vibrio* spp. observed during the first (black line), middle (tan line), and last (red line) six years of study duration at station 70S. Error bars are standard error of the mean.

Time-series analysis, (Fig 4, r^2^ = 0.38) generated a model showing the increasing trend and permitted the forecasting of *Vibrio* spp. in the NRE. The mean monthly *Vibrio* spp. abundance at station 70S was averaged over 6 years, with 3 six-year periods being examined (Fig 5). The periods included 2005–2010, 2007–2012, and 2009–2014, i.e. the first, middle, and last six years of the project. In the last 6 years of the project, significantly more (P<0.0001) *Vibrio* spp. were observed than the first 6 years. This is especially striking as there are two years of data overlap (for 2009 and 2010) that is shared between the first and last 6-year period. The largest difference in means was seen in January, February, and March (1.02 log, 1.17 log, and 1.04 log, respectively). This shift became evident during the routine sampling, as early in the project, *Vibrio* spp. were generally non-detectable at times during the winter, while during the last years of the project, all samples and all seasons, including the coldest months, contained culturable *Vibrio* spp. The 10-year monthly average of total *Vibrio* spp. was calculated for actual (2004–2013), 2-year projection (2006–2015) and 5 years projected (2008–2018, Fig 6). If *Vibrio* spp. abundance increases at a similar rate, that there could be nearly a full log difference in total projected *Vibrio* abundance in every month after 5 years (e.g. in July 2.71 log vs. 3.56 log, Fig 5).

**Fig 6:**
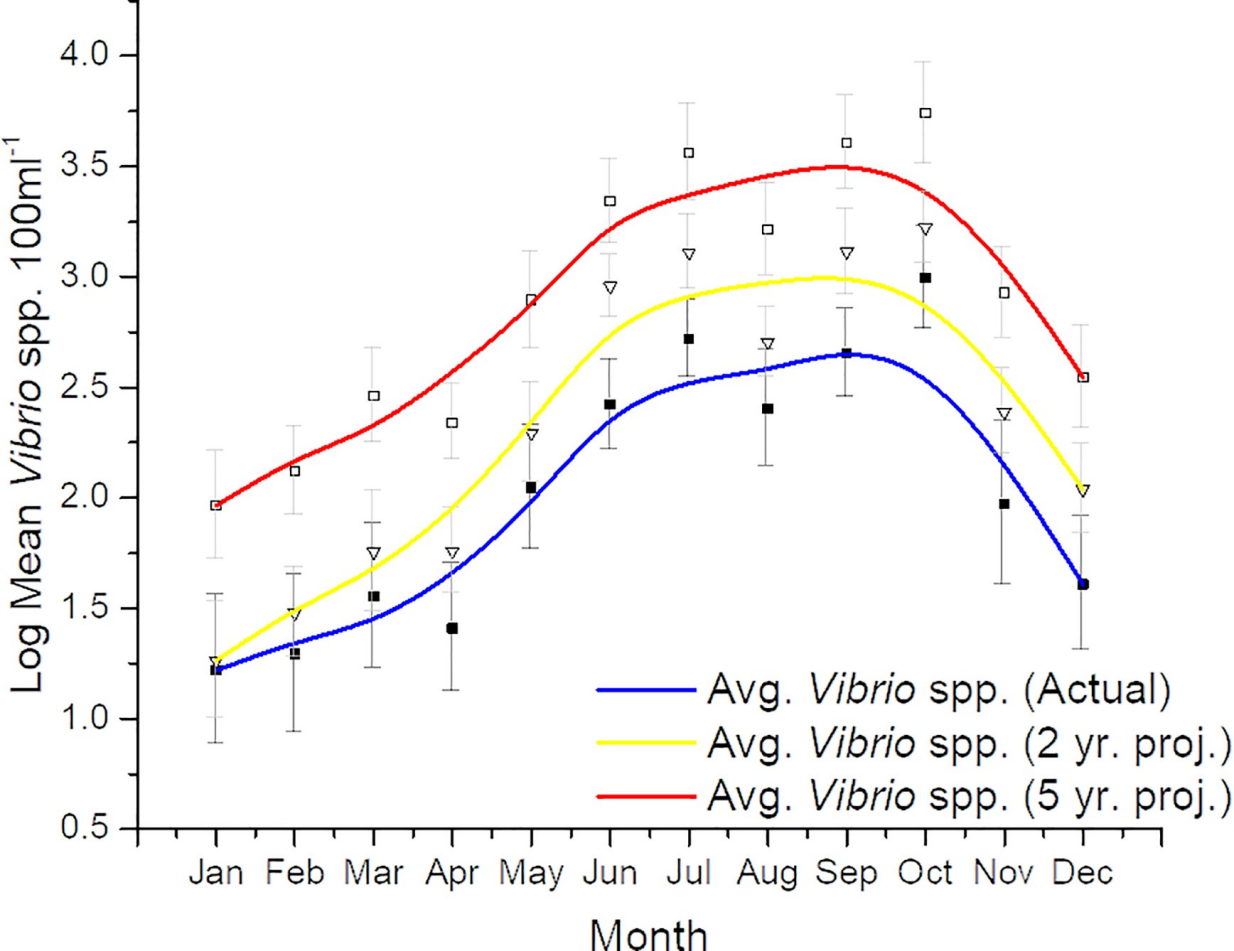
10-year monthly average *Vibrio* spp. abundance (symbols) and 3-month moving average (lines) during the study (blue line), and 2 (yellow line) and 5 (red line) years beyond the study. Error bars are standard error of the means.

### *Vibrio* spp. increases are not uniform

**Fig 7:**
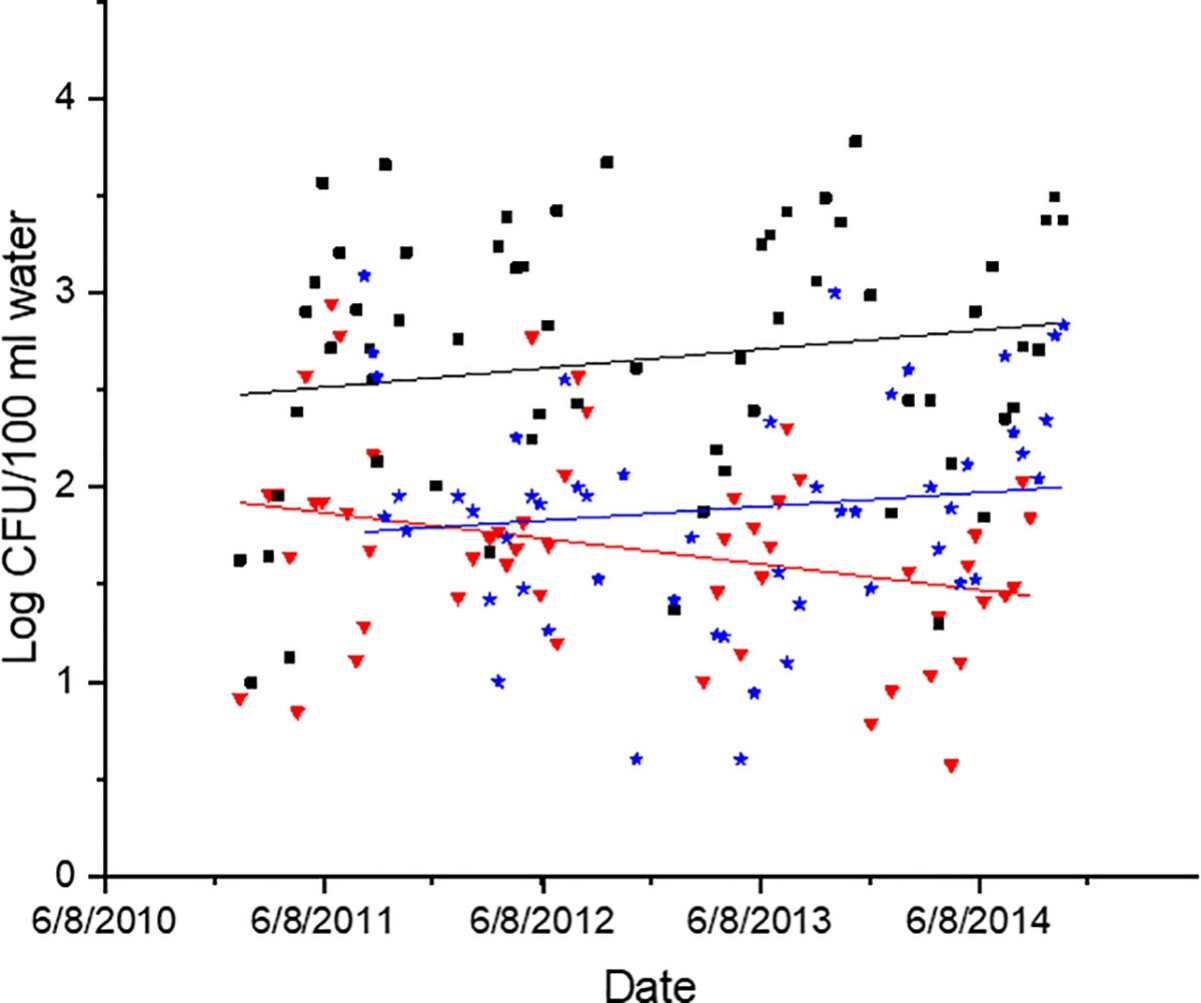
*Vibrio* spp. (black squares), *V*. *vulnificus* (red triangles), and *V*. *parahaemolyticus* (blue stars) concentrations at each sampling date for the last four years of study at station 70S. Lines are the linear regression of the data for *V*. *vulnificus* (red line, r^2^ = 0.1, p<0.05), *V*. *parahaemolyticus* (blue line, p>0.05) and *Vibrio* spp. (black line, p>0.05). Non-detectable samples were removed from this analysis.

Species-specific data on *V*. *vulnificus* and *V*. *parahaemolyticus* was collected the last three years of the study. There was a small but significant linear decrease (R^2^ = 0.10, p<0.05) in *V*. *vulnificus* over those three years while the total *Vibrio* spp. and *V*. *parahaemolyticus* populations did not decrease (Fig 7).

### Correlation of *Vibrio* abundance with measured variables

Spearman’s rank correlation coefficients and significance were calculated for Log *Vibrio*, TSS, TEMP, SAL, DO, DO saturation, pH, TURB, CDOM, POC, PN, DOC, DIC, NOX, NH4, DIN, TDN, DON, PO4, and CHL. A complete list of correlations is in S1 Table, while significant correlations are listed in Table 1. Because several of the measured variables obviously co-vary (e.g. DIN and NOX), the significant variables were used in a principal component analysis. The first 5 principal components (PC) described 83.5% of the variation seen in *Vibrio* spp. abundance (S2 Table). These 5 PC (Table 2) were regressed with Log *Vibrio* spp. abundance stepwise and only PC 1 and 2 were found to have significant effect (p<0.0001) in the model, generating the equation: Log *Vibrio* = 2.09912 + (PC1 * -0.18173) + (PC2 * 0.19563) with r^2^ = 0.42. The eigenvectors with the largest coefficients of PC 1 and PC2 were SAL/CDOM/DIC/NOX/DIN and TEMP/DO/DOC/DON, respectively.

**Table 1:** Spearman’s Rank correlation coefficients and p values of measured variable significantly corrected to log *Vibrio*.

	Corr. coef	p value
**TEMP**	0.41493	< .0001
**SAL**	0.46661	< .0001
**DO**	-0.39972	< .0001
**TURB**	-0.25777	< .0001
**CDOM**	-0.26351	0.002
**DIC**	0.52297	< .0001
**NOX**	-0.54125	< .0001
**DIN**	-0.33622	< .0001
**TDN**	-0.19375	0.008
**DON**	0.22437	0.002
**PO4**	0.23061	0.002
**TC**	0.46306	< .0001

**Table 2:** Eigenvectors of the principal components.

	PC 1	PC 2	PC 3	PC 4	PC 5
TEMP	-0.19248	0.38630	-0.20129	0.26589	-0.40561
SAL	-0.38242	0.05313	0.38318	-0.15641	0.17040
DO	0.04040	-0.47686	-0.21787	-0.25300	0.37175
pH	-0.19842	-0.28107	-0.48587	-0.06252	0.08977
TURB	0.25845	-0.17136	0.07708	0.28147	0.05055
CDOM	0.37154	0.22121	-0.25614	-0.25750	0.11455
DOC	0.29747	0.35773	-0.12827	-0.14252	0.12061
DIC	-0.39715	0.08478	0.34237	-0.07664	0.15437
NOX	0.30235	-0.29391	0.25330	0.31205	-0.25947
NH4	0.21945	0.24779	0.36520	-0.25228	0.31895
DIN	0.39121	-0.16055	0.30898	0.12653	0.02836
DON	0.15450	0.36917	-0.12465	-0.00694	0.10384
TC	-0.09044	0.13514	-0.12392	0.68706	0.65230

### The role of temperature on *Vibrio* abundance in the Neuse River Estuary

**Fig 8:**
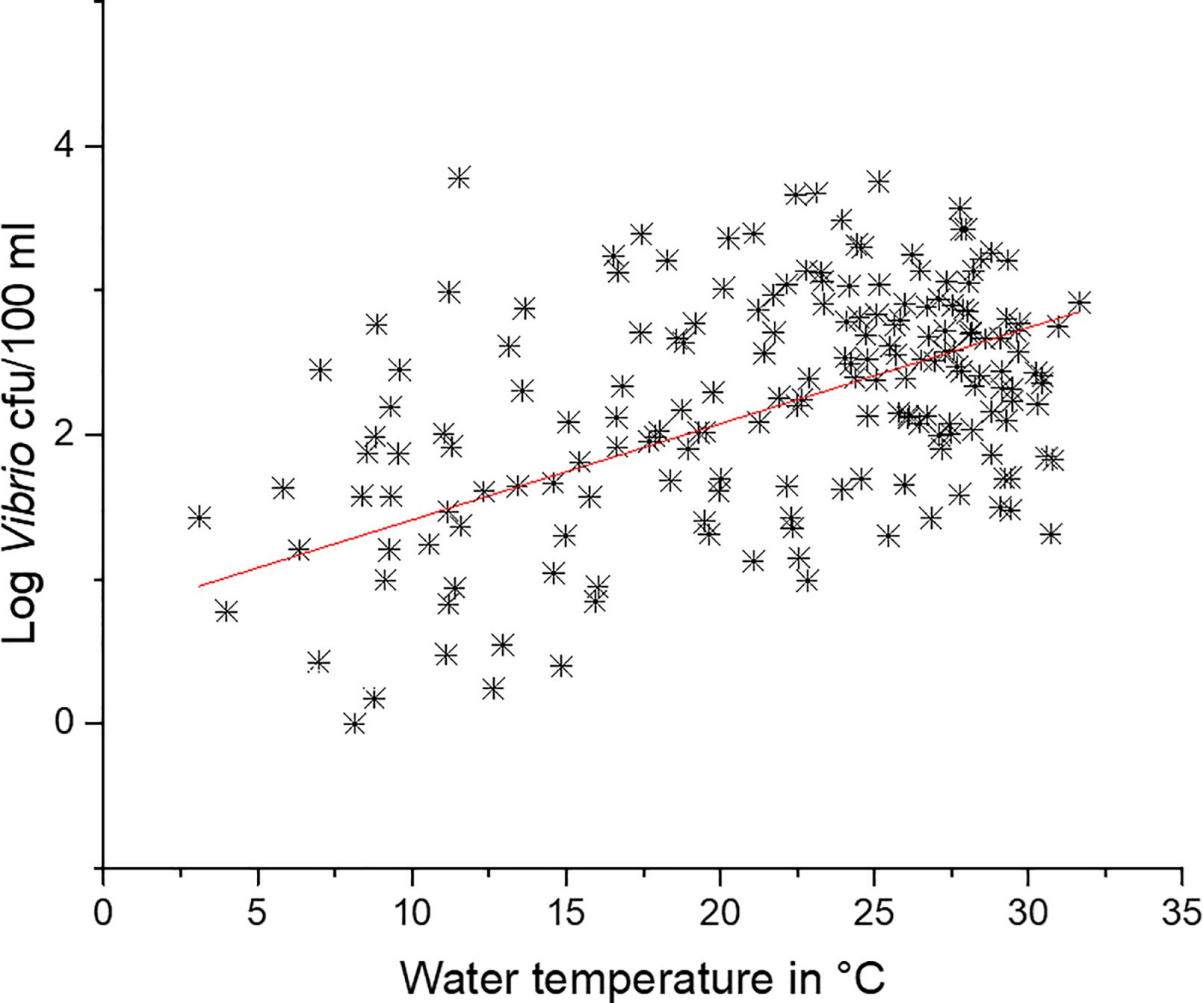
Linear regression of water temperature and log *Vibrio* spp. concentration at station 70S. Regression line = p<0.05 r^2^ = 0.214.

**Fig 9:**
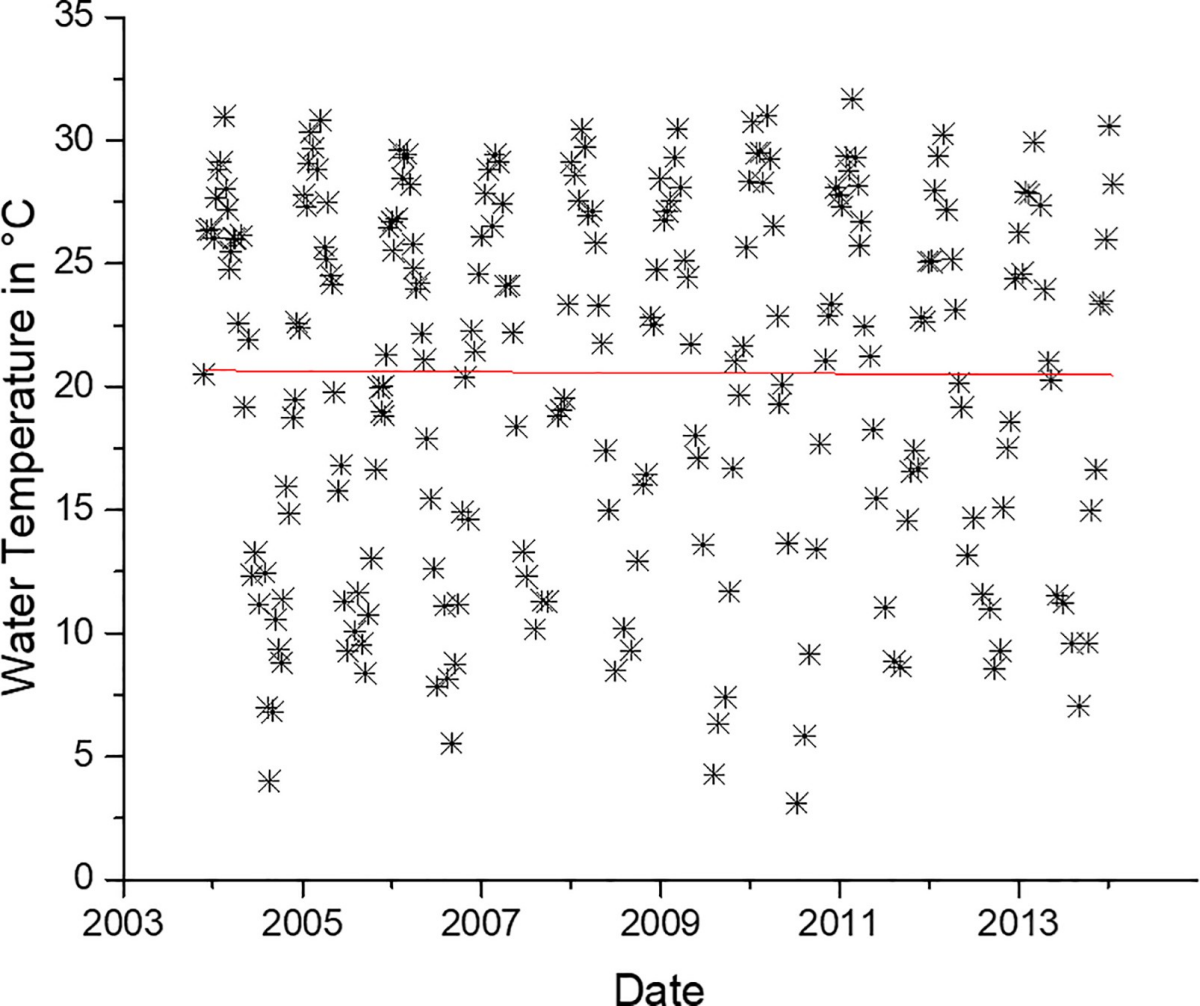
Water temperature at station 70S in the Neuse River Estuary during each sampling date. Slope of regression line is not significantly different than zero (p>0.05).

As has been shown in several other studies at other locations and in the NRE, log *Vibrio* spp. abundance has a significant (p < .05, r^2^ = 0.21) linear relationship with temperature, shown in Fig 8 [3,12,26,30,31,34–37,39,46]. Water temperature trends in the NRE have not changed significantly (p>0.05) during the 10-year study (Fig 9), indicating that an increase in water temperature is not responsible for the increased *Vibrio* spp. abundance.

### The role of salinity on *Vibrio* spp. abundance in the Neuse River Estuary

**Fig 10:**
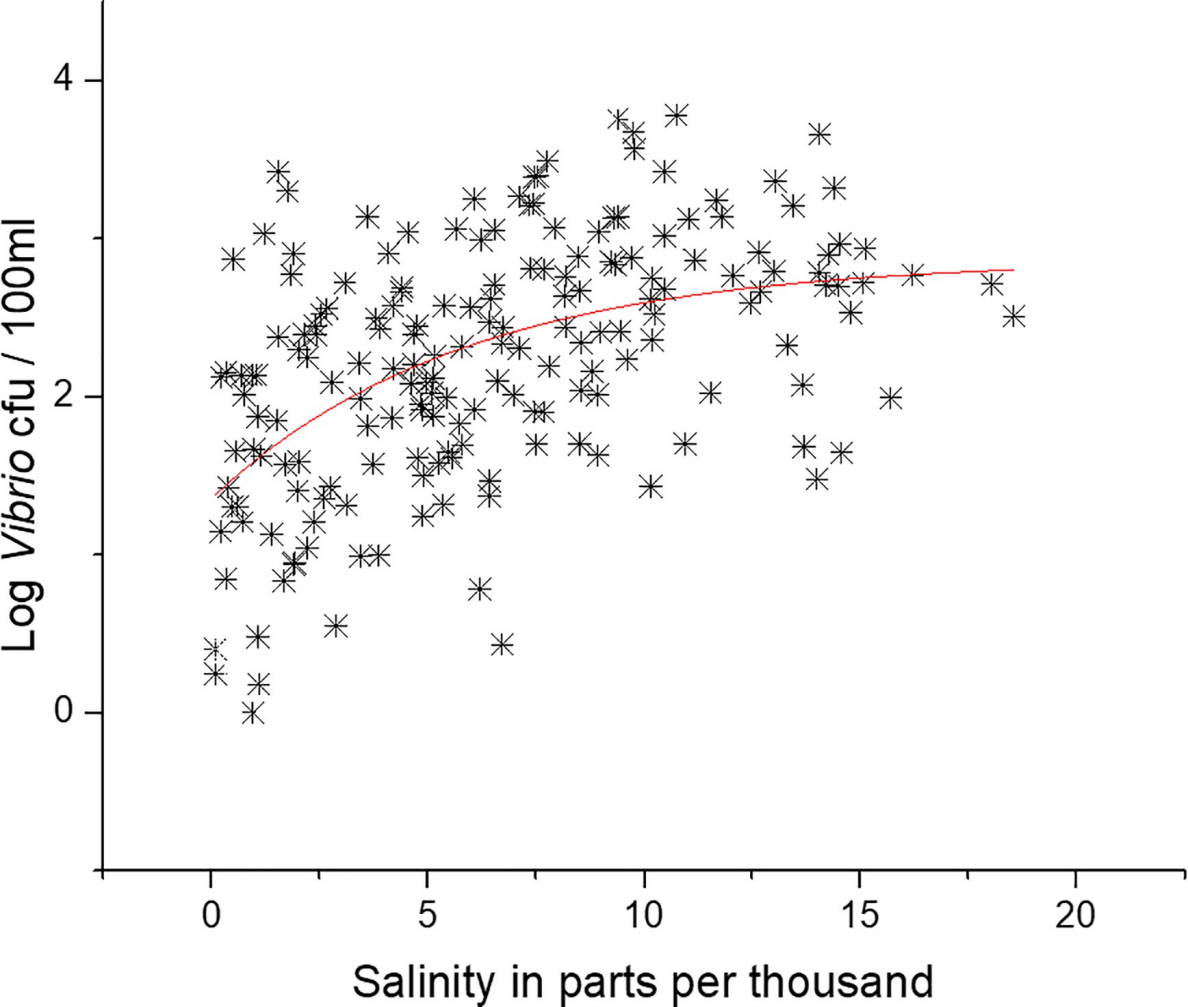
Non-linear regression of salinity and log *Vibrio* concentration at station 70S. Regression line = p<0.05 r^2^ = 0.246.

**Fig 11:**
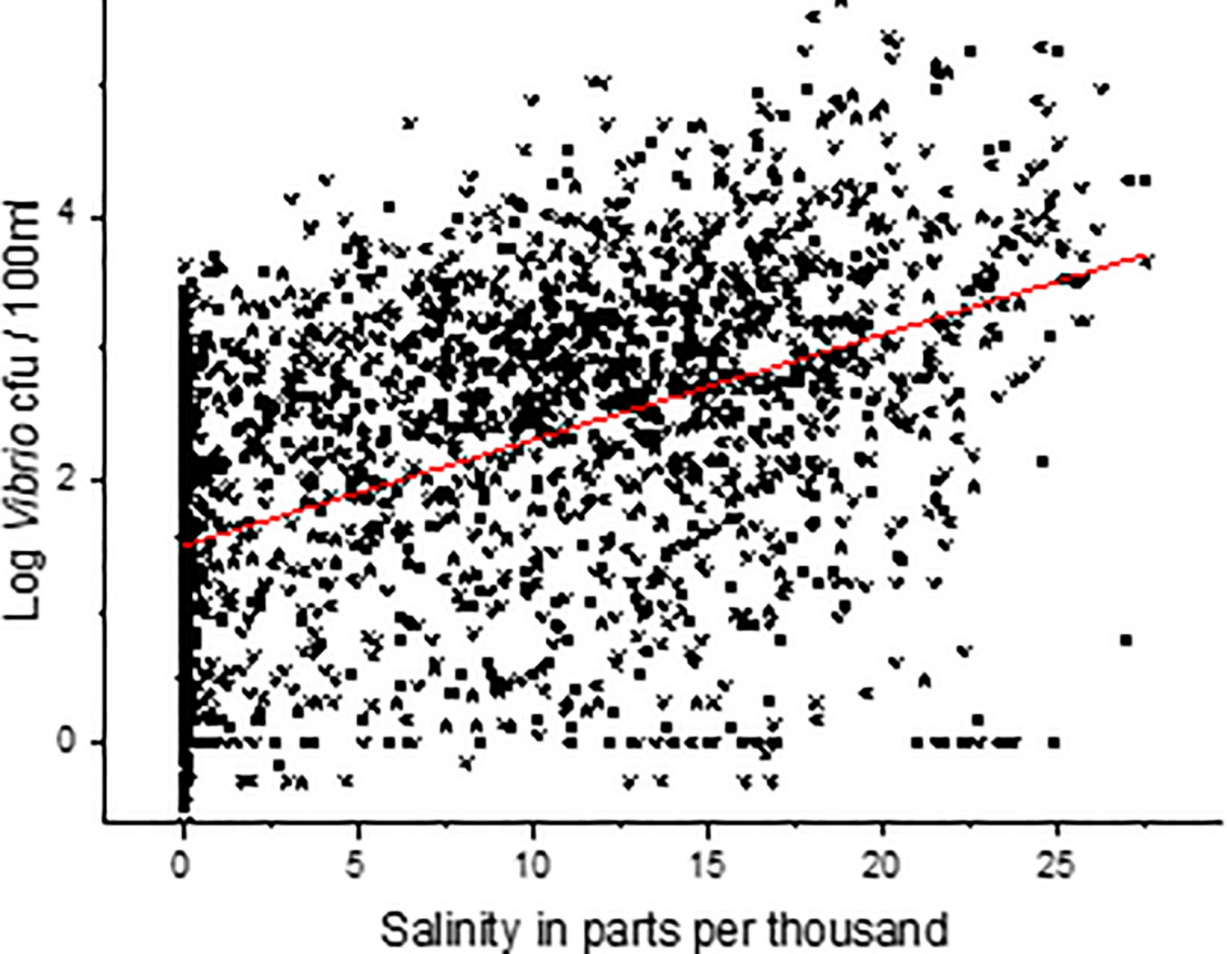
Linear regression of salinity and log *Vibrio* concentration at all stations and depths (N = 2120). Regression line = p<0.05 r^2^ = 0.23.

**Fig 12:**
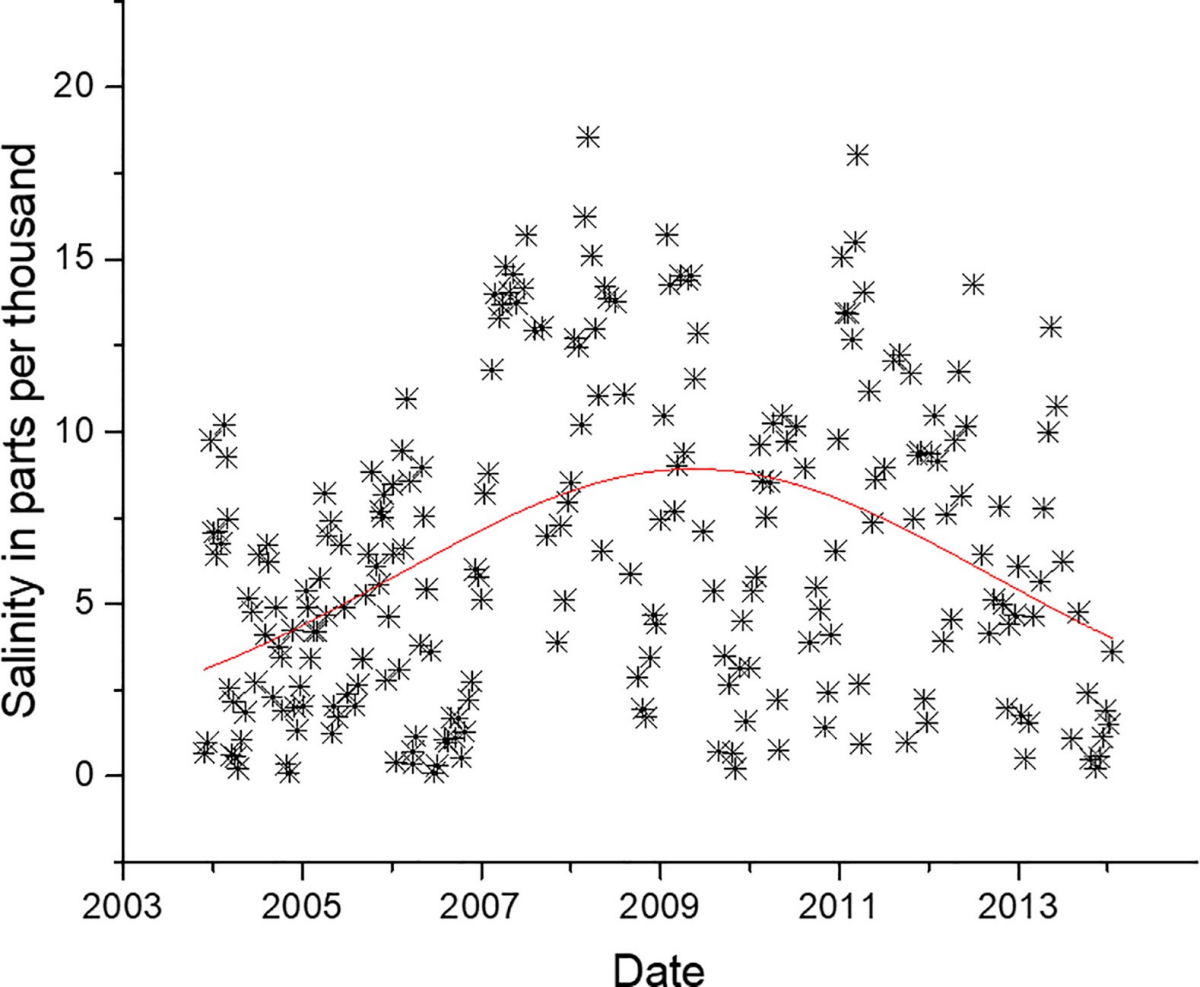
Salinity of station 70S over the course of the study. Red line is LogNormal fit to data, r^2^ = 0.165.

**Fig 13:**
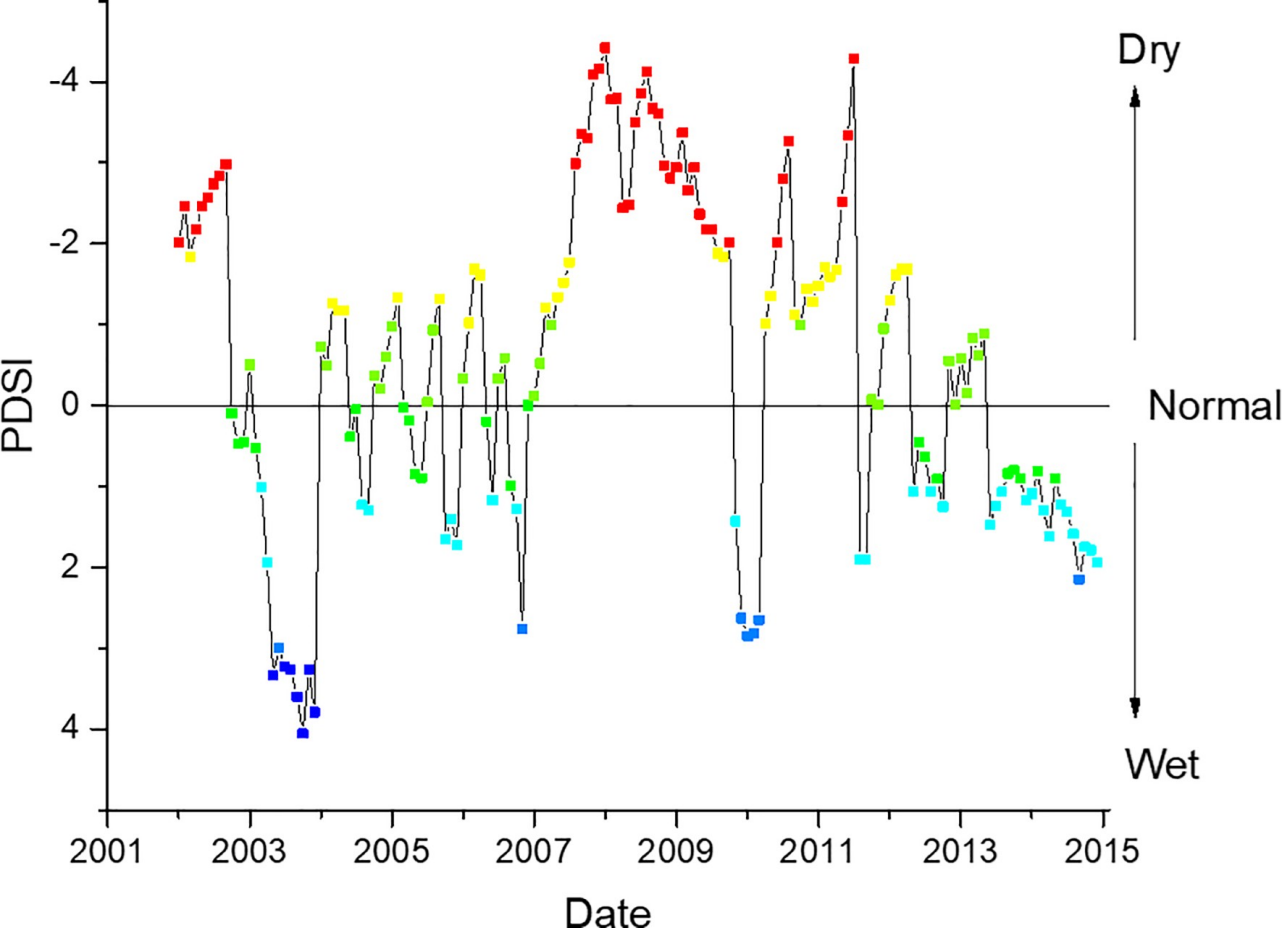
Palmer Drought Severity Index for the Neuse River Estuary during the course of the study. Scale is reversed with negative (dry) values being the topmost part of the graph. Colors added to aid in visualization of drought index severity.

There was a significant non-linear relationship (p<0.05, r^2^ = 0.25) between salinity and log *Vibrio* spp. abundance (Fig 10), which has been reported previously [3,4,26,30,32,34,37,39] in other locations and in the NRE. The non-linear relationship was stronger than a linear relationship (r^2^ = 0.23, S5 Fig), though the maximum salinity recorded at station 70S was 18.56, a permissive salinity for most coastal *Vibrio* spp. When including all the samples from all stations and depths along the entire estuary, the minimum and maximum salinity values are 0 and 27.56‰. When the data from the entire estuary were used in both linear and non-linear regression, the r^2^ values were 0.23 and 0.24, respectively, though visually the linear fit seems more appropriate (Fig 11). Salinity in the estuary has not been increasing, and in fact was at some of the lowest values when *Vibrio* spp. concentrations were the highest. Fig 12 shows the salinity of station 70S over the course of the study (r^2^ = 0.17). The data show that salinity increased during the middle of the study and then decreased towards the end. The salinity data mirrors the precipitation the estuary received during the study period. The freshening of the NRE has been reported previously by Van Dam and Wang [47]. Fig 13 shows the Palmer Drought Severity Index (PDSI), with extreme drought conditions between 2007 and 2011 when NC suffered the worst drought in the history of the state. The PDSI uses precipitation and temperature to estimate relative dryness in a standardized index that ranges from 10 (wet) to– 10 (dry). A return to normal and wet conditions, with a corresponding decrease in estuarine salinity at station 70S, occurred in mid-2011. The drought index increased (wetter conditions) at the same time that increase in *Vibrio* spp. abundance began. This appears to be unrelated to salinity, however, because increased salinity correlates with increased *Vibrio* spp. abundance, but salinity decreased during the *Vibrio* spp. increase.

### Dissolved oxygen and the relationship to *Vibrio* spp. increase

Similar to previous studies [30,31,34–36,46] DO was negatively correlated (Table 1) with *Vibrio* spp. abundance and linear regression showed a weak but significant (p<0.05, r^2^ = 0.10) negative relationship (S1 Fig). While the relationship exists, DO in the NRE did not change significantly over the course of the study (S2 Fig, p>0.05), thus changing DO is not related to the increased abundance.

### The relationship of carbon and nitrogen to *Vibrio* spp. abundance

**Fig 14:**
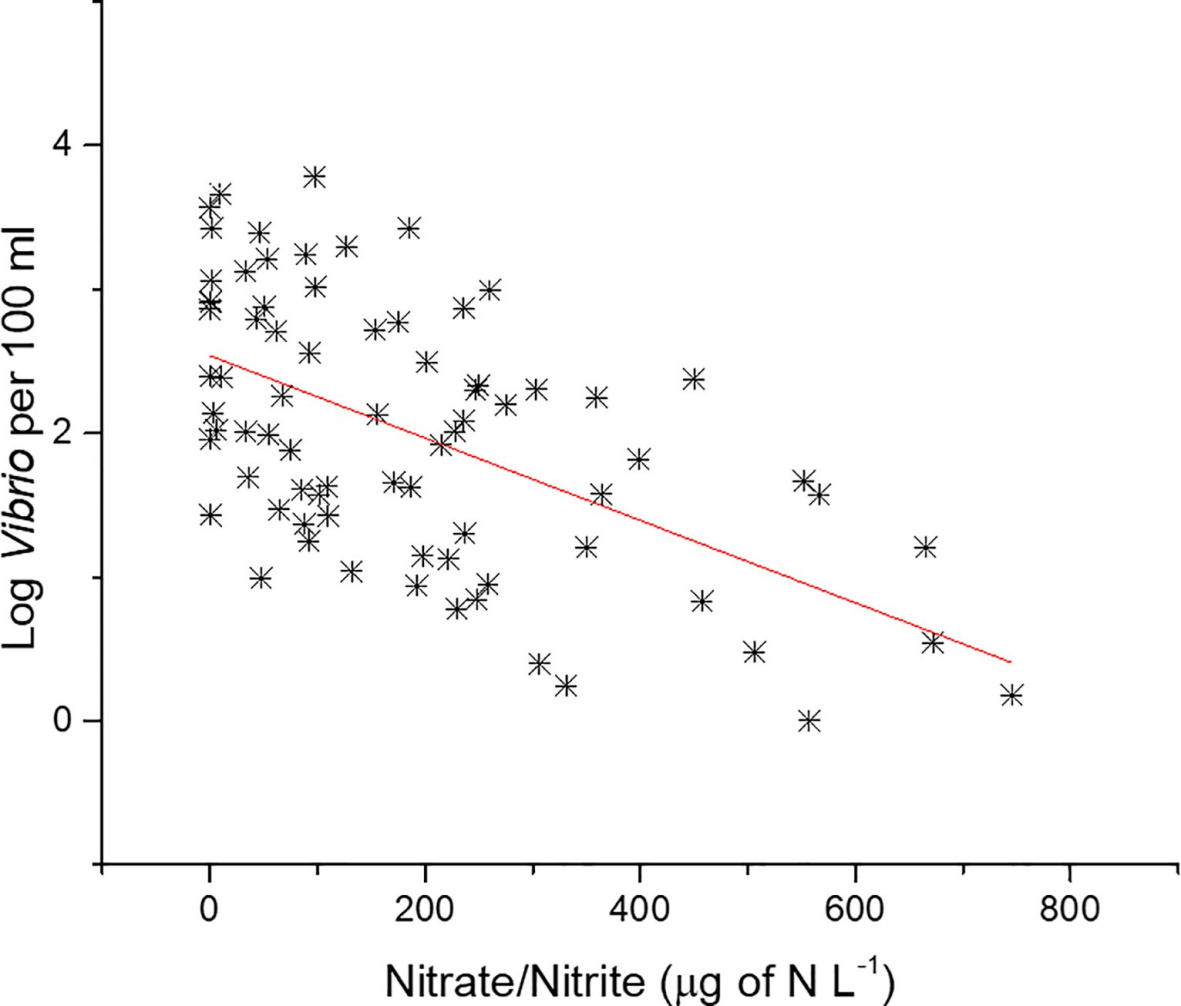
Linear regression of *Vibrio* abundance to NOX at station 70S. Regression line is p<0.05, r^2^ = 0.31.

**Fig 15:**
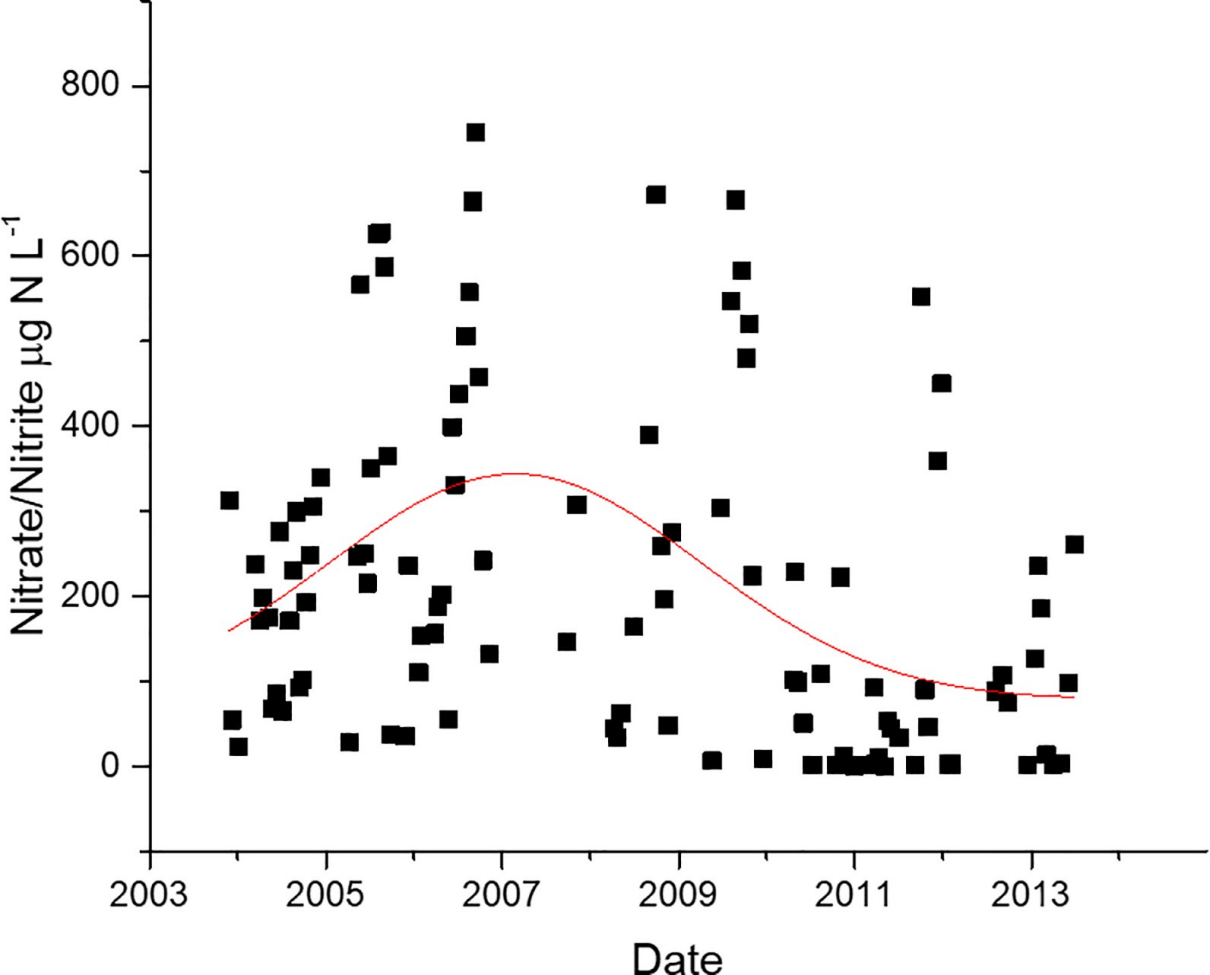
Nitrate and nitrite at station 70S over the course of study. Gauss non-linear fit to data (red line), r^2^ = 0.19.

In the NRE, there was a significant negative correlation between NO_3_/NO_2_ (NOX) and *Vibrio* spp. concentrations (Table 1). Regression analysis shows a significant decrease in *Vibrio* spp. concentrations as the amount of NOX increases (Fig 14, p<0.05, r^2^ = 0.31). In the years of the largest increase in *Vibrio* spp. observed in the NRE, starting in mid-2011, the NOX levels were the lowest, as noted by a Gaussian non-linear fit of the NOX data at station 70S over the course of the study (Fig 15, r^2^ = 0.19). NOX and salinity are negatively correlated, and it was considered that the relationship with *Vibrio* spp. could just be a byproduct. But when salinity decreased in 2011, so did NOX, yet this was the period of the largest *Vibrio* spp. increase. Therefore, it appears that reduced NOX is potentially related to increased *Vibrio* spp. abundance, as lower NOX correlates with larger *Vibrio* spp. concentrations.

**Fig 16:**
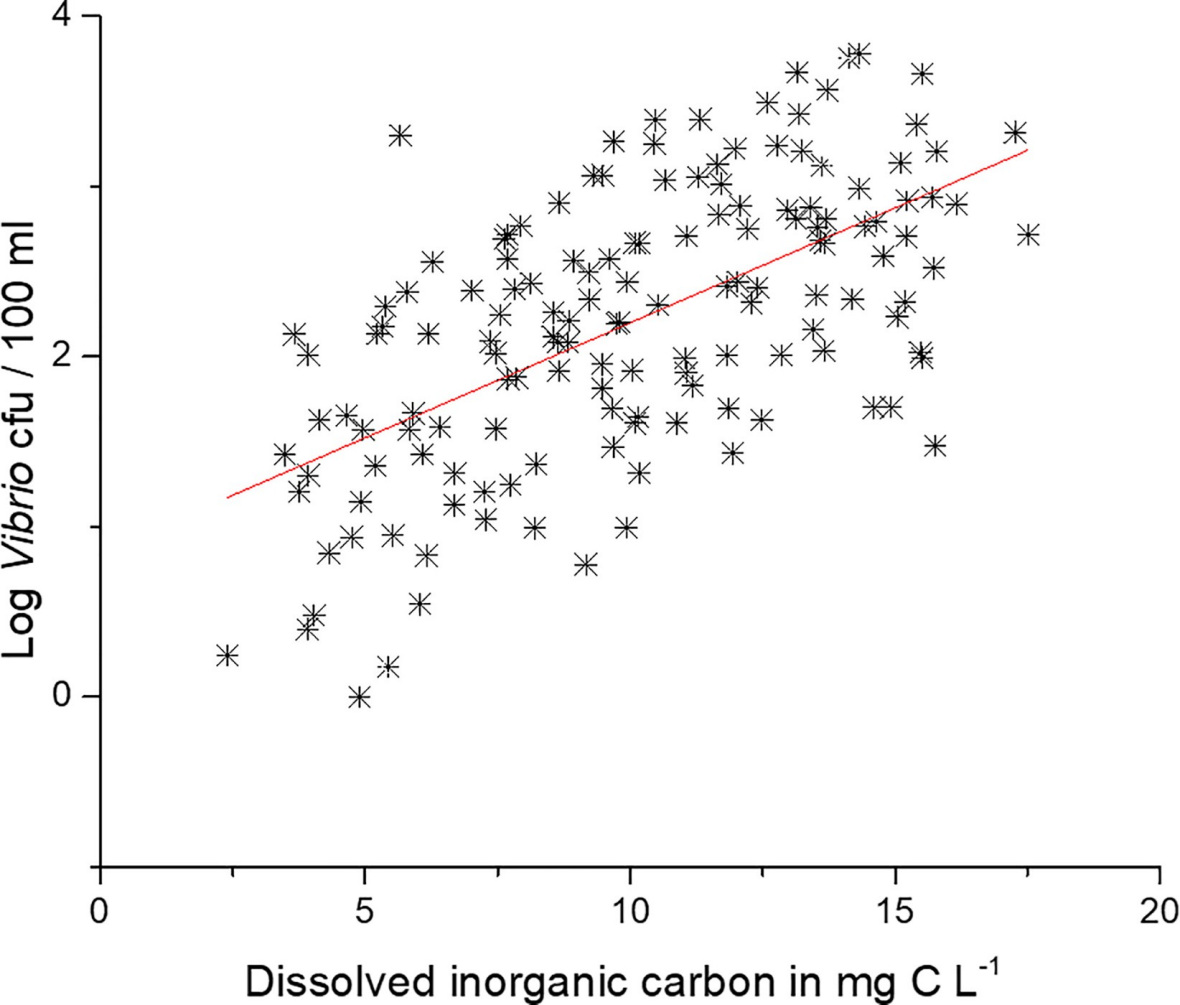
Regression of dissolved inorganic carbon and log *Vibrio* concentration at Station 70S. Red regression line is significant (p<0.05, r^2^ = 0.37).

**Fig 17:**
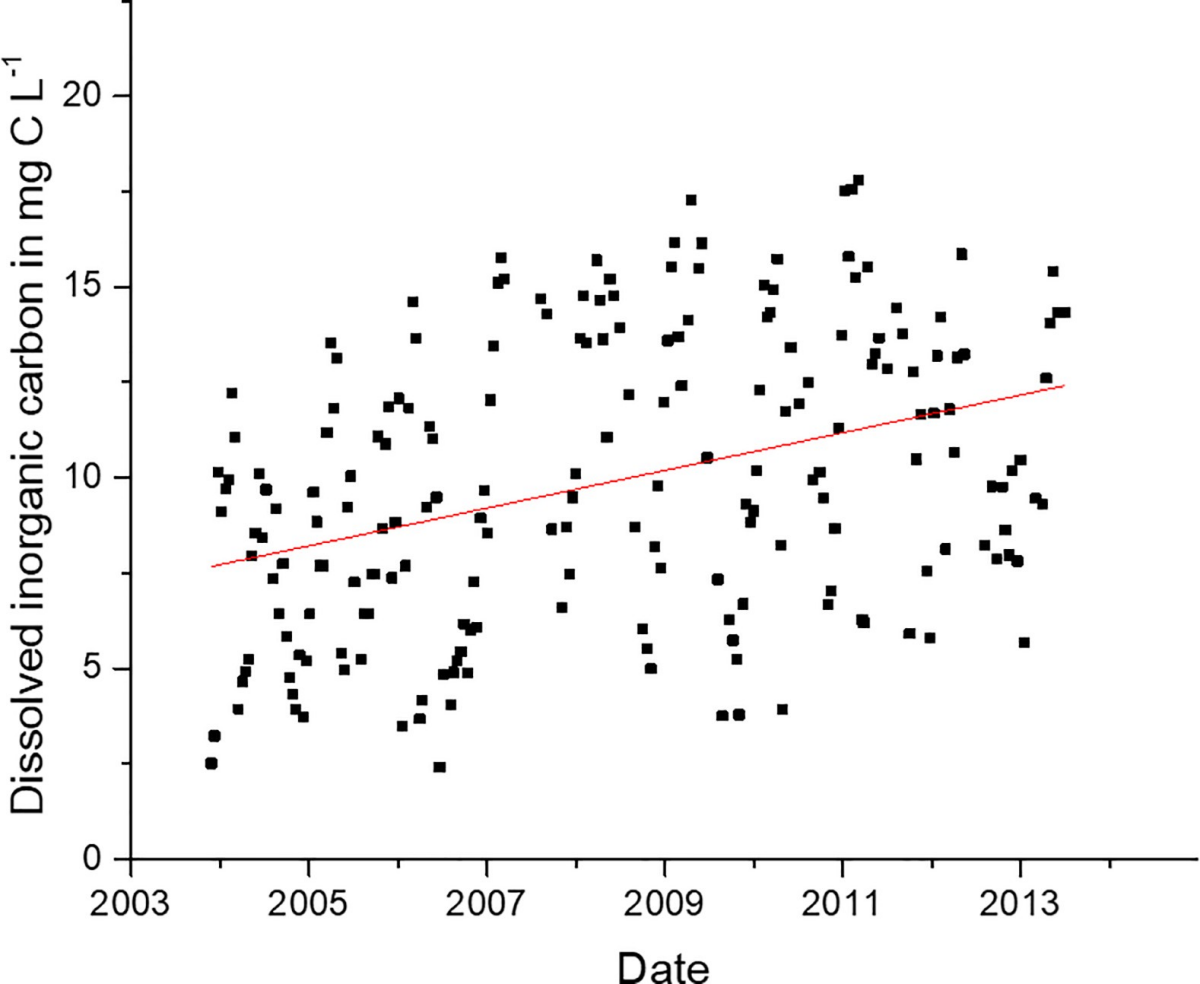
Dissolved inorganic carbon at station 70S recorded during the duration of the study. The slope of the linear fit of data (red line) is significantly different from zero (p<0.05, r^2^ = 0.12).

A significant correlation (Table 1) and linear regression (p<0.05, r^2^ = 0.37) of DIC and *Vibrio* spp. concentrations is shown in Fig 16. A linear fit of DIC at station 70S over the course of the study reveals a significantly higher trend (p<0.05, r^2^ = 0.12, Fig 17). DIC and salinity are strongly correlated, but as with NOX, the changes in salinity in the estuary do not appear to correspond with the changes in *Vibrio* spp. Thus, it is likely the increase in DIC over time in the NRE could be a potential contributor to the increased *Vibrio* spp. abundance that was observed.

Regression analysis revealed that lower DIN concentrations correlate with increased *Vibrio* spp. abundance (p<0.05, r^2^ = .23, S3 Fig). DIN concentrations did not change significantly over the course of the study (p>0.05, S4 Fig), an indication that DIN was not involved in the increase in *Vibrio* spp.

### Five year predicted vs. actual sample concentrations

Utilizing the same methodology, additional samples were collected in late September to late October of 2018. The 5-year prediction in Fig 6 was compared to the actual data collected in 2018 and is displayed in Table 3. The values predicted for 5 years after the last decadal sample were higher than the actual samples collected (Table 3), yet strikingly, all samples collected in 2018 were greater than the historical samples, with one being nearly one log greater (Table 3).

**Table 3:** The historic average (during 2003–2013), the predicted values, actual values, and differences from historic and predicted concentrations of *Vibrio* from station 70S in the NRE collected during 2018.

Sample Date	Historic Value	Predicted Value/100ml	Actual Value/100ml	Increase from Historic	Difference from predicted
Sept 26, 2018	380	2344	730	350	1614
Oct 8, 2018	275	2188	1200	925	988
Oct 12, 2018	182	1513	231	49	1282
Oct 30, 2018	120	912	740	620	172

## Discussion

This is the longest *Vibrio* spp. monitoring program that has taken place in the State of North Carolina, and perhaps along the east coast of the United States. *Vibrio* spp. were routinely monitored for over ten years. The NRE, the site of the monitoring program, is part of the Albemarle-Pamlico Estuarine System (Fig 1). The NRE experiences large seasonal variability in nutrient concentrations [48] and is affected by anthropogenic inputs, both urban and agricultural. The estuary is also heavily used, both commercially and recreationally, and is part of the Intracoastal Waterway. The NRE is undergoing eutrophication, driven in part by urban expansion, agricultural runoff, and high degree of livestock operations occurring in the watershed [41,49]. Thus, factors driving the health of this important estuarine ecosystem are changing making an understanding of the dynamics of bacterial populations important to study. This continuous monitoring effort revealed that *Vibrio* spp. concentrations appear to be increasing in the NRE in eastern NC (Fig 3). Yearly averages show that *Vibrio* spp. means tended to increase one year, lower the next, and then exhibit an even larger increase the following year (Fig 2). A seasonal ARIMA model shows that the increase was heaviest starting in 2011, and in 2012 throughout the rest of the study, *Vibrio* spp. exhibited continuous detection, even in the winter months (Fig 4). The increase of winter *Vibrio* spp. is especially noticeable when monthly averages from the beginning, middle, and ending of the monitoring program are compared (Fig 5). The largest increases, based on monthly averages, were in the cold winter months. This indicates that the seasonal reduction in either live or culturable *Vibrio* spp., that had been considered normal, is no longer as pronounced. This has important ramifications for species-specific shifts in the total *Vibrio* spp. population, and especially important ramifications for winter-dominant commercial shellfish harvest. *Vibrio* spp. have been thought to either enter the viable-but-non-culturable state or overwinter in the sediments, but they are detectable year-round now [35,50–52]. Interestingly, not all *Vibrio* species are behaving in the same fashion. Individual species data were only collected the last 4 years of the study including *V*. *vulnificus* and *V*. *parahaemolyticus*. *V*. *vulnificus* showed a significant decrease during that period while the total *Vibrio* spp. and *V*. *parahaemolyticus* populations did not (Fig 7). This seems to indicate that some species are becoming more abundant, and that the species makeup of the *Vibrio* spp. population may be in flux. Shifts in water column conditions have been shown in the NRE previously to affect certain *Vibrio* species in differing manners, such as was seen after the prolonged drought around 2007 (Fig 13) caused *V*. *vulnificus* to nearly disappear from the estuary while more salt tolerant species were thriving [53]. Similar effects have been reported in the Gulf Coast [54]

Increased *Vibrio* spp have been reported in various coastal areas throughout the world. Vezzulli et al [55], using a continuous plankton recording device, showed that *Vibrio* spp. abundance was increasing in parts of the North Atlantic and North Sea. They concluded these increases were due to rising sea water temperatures. A report by Martinez-Urtaza et al [56] describes some pathogenic *Vibrio* species increasing in Peru, Alaska, and the Gulf of Mexico due to warmer water temperatures. Other reports by Baker-Austin and others describe emerging *Vibrio spp*. related infections occurring in novel areas, such as Sweden and Finland, caused by anomalous increases in water temperature [7,24]. Interestingly, the increase observed in the NRE was not accompanied with anomalous temperature shifts and the water temperature during the decade of the study has not changed significantly (Fig 9). Temperature typically has the strongest influence on *Vibrio* spp. concentrations in natural environments, including in the NRE, but evidence suggests that water temperature on the coast of NC may actually be decreasing [55]. Recently, the effects of temperature were examined on the abundance of *V*. *vulnificus* in South Carolina [57]. *V*. *vulnificus* abundance is normally strongly dependent on temperature [50,58,59] yet this study found that an increase in *V*. *vulnificus* was not obviously related to temperature, but rather to sea level rise. Thus, other factors were investigated that might be related to the increase in *Vibrio* spp. in the NRE.

Salinity is commonly the second most influential factor in determining the abundance of coastal *Vibrio* spp, and this is true in NC (Figs 9 and 10) as well [3,4,26,30,32,34,36,37,53,55]. Salinity in the NRE at this study location rose, peaked, and then later fell (Fig 12) during the decadal study. This shift in salinity in the NRE corresponded with a record-breaking drought [53], the most severe in NC history, that began in 2007 and persisted until 2011 (Fig 13). During this drought, when salinity levels were elevated, some specific species of *Vibrio* (i.e. *V*. *vulnificus*) declined to the point of being nearly undetectable, while others that were more salt resistant, including *V*. *mediterranei* and *V*. *coralliilyticus*, were detected instead [53,60]. Even though salinity and *Vibrio* spp. abundance is strongly correlated, the increase in *Vibrio* spp. in the NRE coincides with an overall decrease in estuarine salinity seen from 2010 through the end of the study, probably as related to freshwater discharge and groundwater height due to precipitation (Fig 12). While short-term increases in salinity do promote higher *Vibrio* spp. concentrations, the longer-term shifts in salinity do not appear to have this effect on the trend overall. Thus, the two biggest contributing environmental factors that have been previously used to predict estuarine *Vibrio* spp. abundance, i.e. salinity and temperature, appear to not be significantly involved in the decadal increase of *Vibrio* spp. observed in the NRE. Thus, it is evident that there is more going on in the NRE that is driving this increase.

Few studies have looked at the correlation between NO_3_ or NO_2_ (NOX) and *Vibrio* spp. A study by Asplund et al [30] found a slight positive correlation with NO_3_ and *Vibrio* spp. *Vibrio* spp. abundance was found to increase with reduced concentrations of NOX in the NRE (Fig 14). There was a marked decrease in NOX in the same years as the largest increases in *Vibrio* spp. were observed (Fig 15). Reductions or limiting amounts of NOX typically result in reduced phytoplankton concentrations (i.e. Chla). Overall, phytoplankton blooms were reported to have been stunted during the study period, attributed to the reduced nitrogen availability, even though there were specific periods with heightened concentrations of specific phytoplankton types [41]. *Vibrio* spp. are able to reduce nitrate to nitrite or ammonia and are often the dominant nitrate-reducing group [61,62]. These are some of the highest energy yielding processes, but typically only in anoxic environments [63]. While it could be possible that the increase in *Vibrio* spp. in the NRE could have resulted in a reduction in the NOX recorded, station 70S did not experience anoxic conditions (S2 Fig) and the level of ammonia did not significantly change over the course of the study (S7 Fig), so this remains speculative. Combined NOX was significantly correlated with CHL, which is a proxy for phytoplankton (S1 Table). There was very slight but significant increase in CHL (p<0.05, r^2^ = 0.03, S6 Fig) over the course of the study. The reduction in NOX could be associated with increased phytoplankton activity as well. Additionally, it has been shown that a reduction in flow will result in a reduction of NOX, because of less runoff for example [42]. In this study, however, the decrease in NOX occurred during a period of increased precipitation, and thus greater flow, suggesting that the NOX decrease was not caused by a lack of input into the system. The lack of correlation of *Vibrio* abundance with CHL (S1 Table) appears to indicate that the increase in *Vibrio* spp. in the system is not associated with phytoplankton abundance.

Increases in DIC had a strong connection with increases in *Vibrio* spp. as shown in Fig 16. The increase in *Vibrio* spp. coincides with an increase in DIC in the NRE (Fig 17). This increase in DIC could be the result of increased photochemical oxidation of DOC, but DOC changes at the site correspond more with rainfall than with *Vibrio* spp. (S8 Fig). Van Dam and Wang [47] showed that the pH of NRE during this time period showed a decrease. DIC does correlate strongly with salinity, but over the course of the study DIC increases in the NRE while salinity only increased in the drought years, followed by decrease (Fig 12). Thus, there is a decoupling of DIC with salinity. The increase in *Vibrio* spp. could potentially be the cause the of increased DIC. The combined increased of DIC with the decrease in NOX is indicative of there being more respiration versus photosynthesis, and indeed after the drought of 2007, and through 2009, primary production was reduced in the NRE [41]. This, however, remains speculation in such a complicated system.

Five years after the last sample of the study was taken, four additional samples were collected at the same location using the same methodology. All four samples contained *Vibrio* spp. concentrations that were greater than the average from the same time during the 10-year study (Table 3). While none of these samples reached the values predicted by the ARIMA model, they are all within the 95% confidence of the model. This appears to indicate that the increase in *Vibrio* spp. observed during 2003–2013 in the NRE is either holding steady or continuing to increase.

The increase in *Vibrio* spp. concentrations in the NRE would, at first glance, not seem unusual as *Vibrio* spp. appear to be increasing in coastal areas worldwide. What makes this increase remarkable is that it does not coincide with increases in temperature, which is the most commonly cited reason for increase in other reports. Furthermore, the second and third most cited influence on *Vibrio* spp. abundance, salinity and dissolved oxygen, respectively, also do not appear to be involved. Measured DO has remained unchanged, over time, while salinity has decreased, which should be associated with a subsequent decrease in *Vibrio* spp. Other factors, including NOX and DIC were found to have correlated with the change in *Vibrio* spp., but are likely a result of the increased bacterial population, rather than a cause. Metagenomic analysis of these samples is being conducted and may offer other explanations of this phenomenon.

## Supporting information

S1 Fig Linear regression of *Vibrio* abundance to dissolved oxygen at station 70S. Regression line is p<0.05, r^2^ = 0.1.
(TIF)

S2 Fig Dissolved oxygen at station 70S during the study. Slope of regression line (red) is not significantly different than zero (p>0.05).
(TIF)

S3 Fig Dissolved inorganic nitrogen vs. log *Vibrio* abundance at station 70S. Red regression line is significant (p<0.05, r^2^ = .23).
(TIF)

S4 Fig Dissolved inorganic nitrogen at station 70S during the study. Slope of regression line (red) is not significantly different than zero (p>0.05).
(TIF)

S5 Fig Linear regression of salinity and log *Vibrio* concentration at station 70S. Regression line = p<0.05 r^2^ = 0.230.
(TIF)

S6 Fig Chlorophyll A at station 70S over time during the study. Red line is linear regression (p<0.05, r^2^ = 0.03).
(TIF)

S7 Fig Ammonia at station 70S over time during the study. Red line is linear regression (p>0.05, r^2^ = -0.005).
(TIF)

S8 Fig DOC at station 70S over time during the study. Red line is 5 order polynomial regression (p<0.05, r^2^ = 0.06).
(TIF)

S1 Table R^2^ values of Spearman correlations. Green Highlights indicated significant (p<0.05) correlations.
(DOCX)

S2 Table Eigenvalues and variance for each principal component. (DOCX)
