# Supplementary figures and images for: Decadal monitoring reveals an increase in Vibrio spp. concentrations in the Neuse River Estuary, North Carolina, USA
Source: PLoS One. 2019 Apr 23;14(4):e0215254. doi: 10.1371/journal.pone.0215254 (PMC6478372; doi:10.1371/journal.pone.0215254)

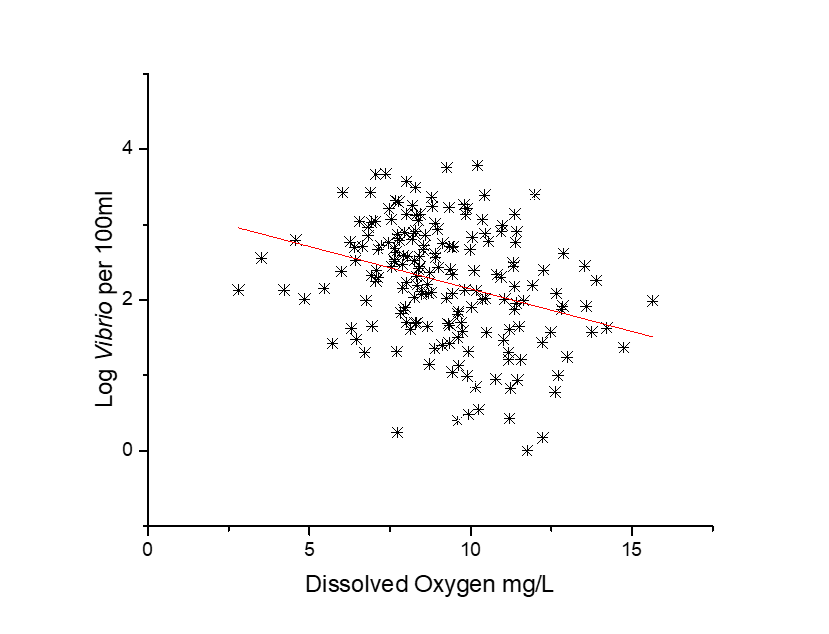

Supplement: S1 Fig — Regression line is p<0.05, r2 = 0.1. (TIF) [file pone.0215254.s001.tif]

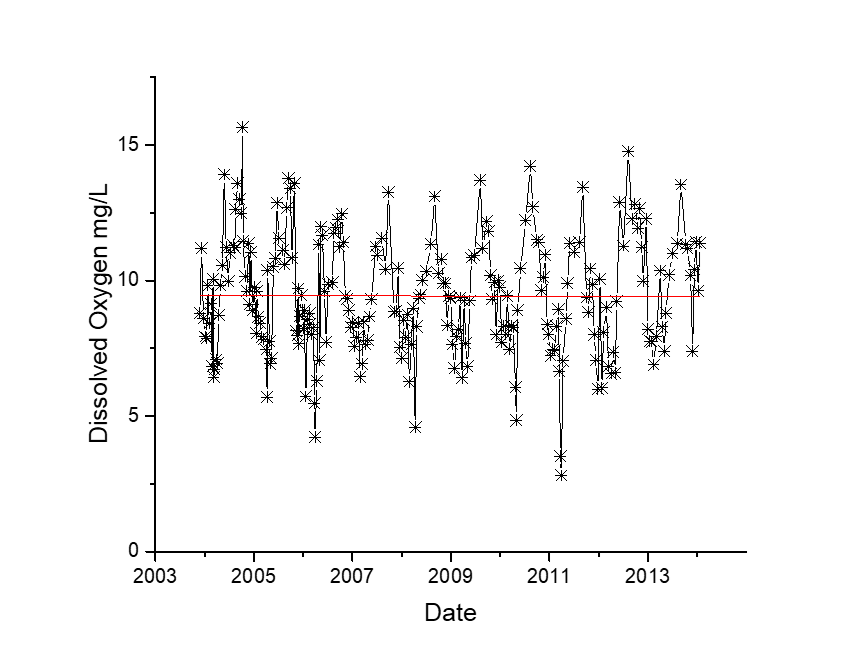

Supplement: S2 Fig — Slope of regression line (red) is not significantly different than zero (p>0.05). (TIF) [file pone.0215254.s002.tif]

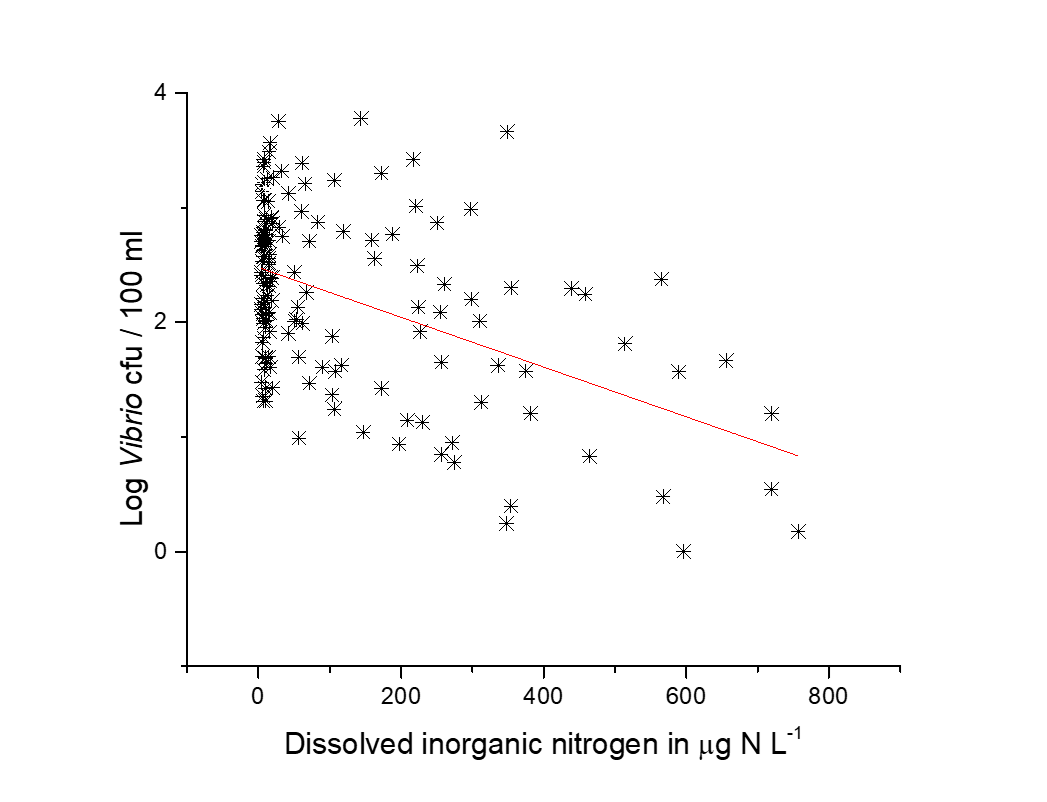

Supplement: S3 Fig — Red regression line is significant (p<0.05, r2 = .23). (TIF) [file pone.0215254.s003.tif]

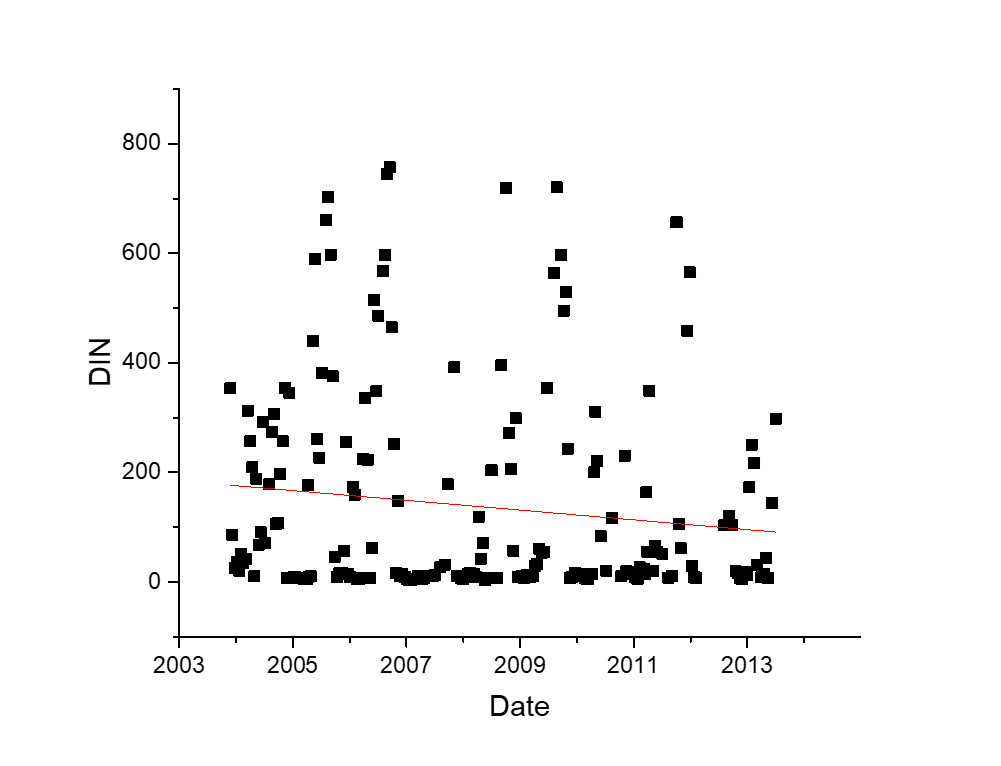

Supplement: S4 Fig — Slope of regression line (red) is not significantly different than zero (p>0.05). (TIF) [file pone.0215254.s004.tif]

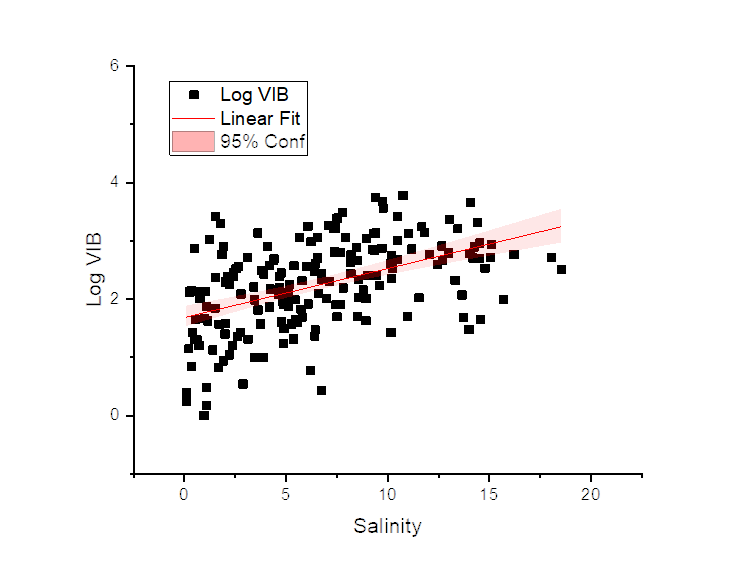

Supplement: S5 Fig — Regression line = p<0.05 r2 = 0.230. (TIF) [file pone.0215254.s005.tif]

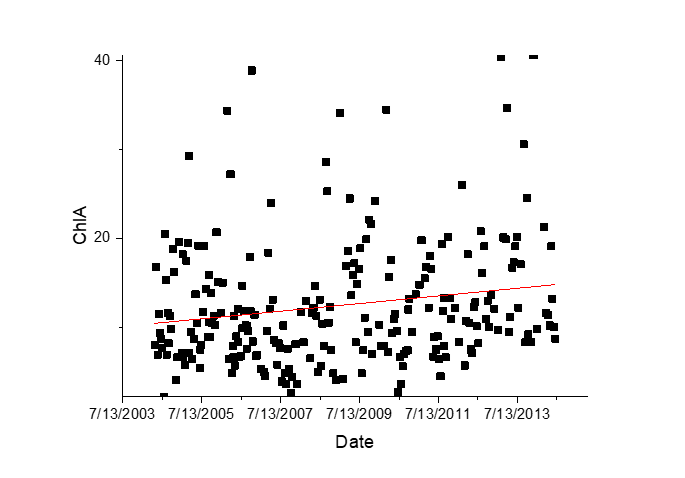

Supplement: S6 Fig — Red line is linear regression (p<0.05, r2 = 0.03). (TIF) [file pone.0215254.s006.tif]

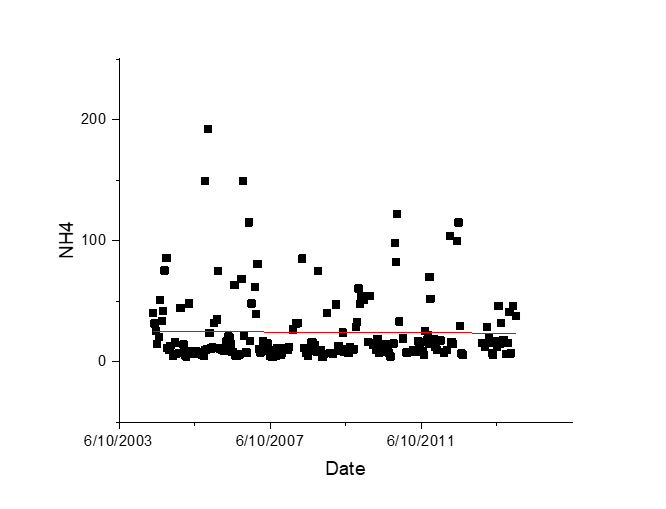

Supplement: S7 Fig — Red line is linear regression (p>0.05, r2 = -0.005). (TIF) [file pone.0215254.s007.tif]

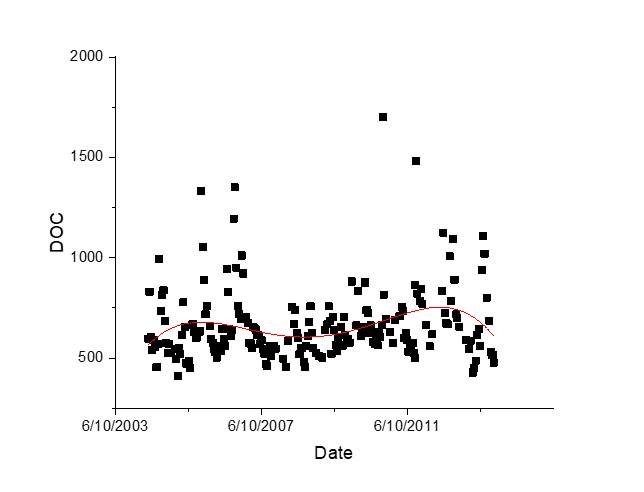

Supplement: S8 Fig — Red line is 5 order polynomial regression (p<0.05, r2 = 0.06). (TIF) [file pone.0215254.s008.tif]

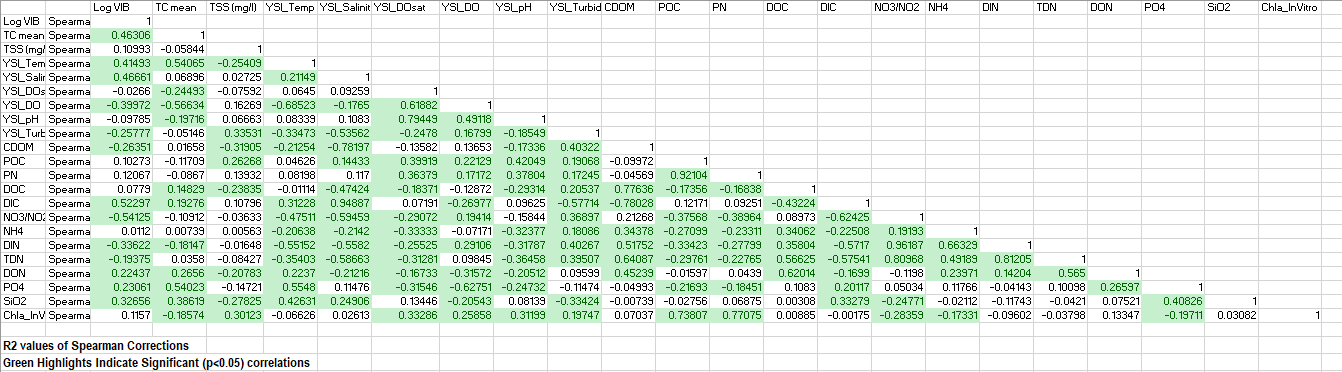

Supplement: S1 Table — Green Highlights indicated significant (p<0.05) correlations. (DOCX) [file pone.0215254.s009.docx]
