# Supplementary material for: Decadal monitoring reveals an increase in Vibrio spp. concentrations in the Neuse River Estuary, North Carolina, USA
Source: PLoS One. 2019 Apr 23;14(4):e0215254. doi: 10.1371/journal.pone.0215254 (PMC6478372; doi:10.1371/journal.pone.0215254)
Supplement: S2 Table — (DOCX) [file pone.0215254.s010.docx]

| Principal Component | Eigenvalue | Percent Variance | Cumulative Percent Variance |
| --- | --- | --- | --- |
| 1 | 4.6186 | 35.528 | 35.528 |
| 2 | 2.9999 | 23.076 | 58.604 |
| 3 | 1.3635 | 10.489 | 69.093 |
| 4 | 1.0993 | 8.456 | 77.549 |
| 5 | 0.7833 | 6.025 | 83.574 |
| 6 | 0.7555 | 5.812 | 89.385 |
| 7 | 0.6277 | 4.829 | 94.214 |
| 8 | 0.4431 | 3.409 | 97.623 |
| 9 | 0.2274 | 1.749 | 99.372 |

Supplementary Table S2: Eigenvalues and variance for each principal component
